# Refining LNA safety profile by controlling phosphorothioate stereochemistry

**DOI:** 10.1371/journal.pone.0232603

**Published:** 2020-06-12

**Authors:** Erik Daa Funder, Nanna Albæk, Annie Moisan, Sabine Sewing, Troels Koch

**Affiliations:** 1 Roche Innovation Center Copenhagen A/S, Hørsholm, Denmark; 2 Roche Innovation Center Basel, Basel, Switzerland; University of Helsinki, FINLAND

## Abstract

Drug discovery with phosphorothioate oligonucleotides is an area of intensive research. In this study we have controlled the stereochemistry of the phosphorothioate backbone of LNA oligonucleotides to investigate the differences in safety profile, target mRNA knock down, and cellular uptake *in vitro*. The study reveals that controlling only four stereocenters in an isomeric phosphorothioate mixture can improve the therapeutic index significantly by improving safety without compromising activity.

## Introduction

Locked nucleic acid (LNA) has over the last fifteen years been intensively used in RNA therapeutics. [1] The key advantage of LNA is the high affinity that LNA nucleosides bring against target RNA when incorporated into oligonucleotides. This higher affinity translates into higher potency for RNA targets for nearly any LNA oligonucleotide composition and design. [2, 3] A widely used design is the gapmer in which 5’-/3’-segments of LNA nucleotides are flanking a central DNA nucleotide segment. When gapmers hybridize to a messenger RNA (mRNA) the DNA/RNA hybrid duplex recruits the enzyme Ribonuclease H (RNase H) which degrades the RNA strand of mRNA/DNA hybrids, [4] altering the protein expression of the mRNA. [5, 6] For most LNA oligonucleotide designs used in RNA targeting applications (i.e. RNA therapeutics) the internucleoside phosphates are replaced by phosphorothioates. [7, 8, 9] This backbone modification is crucial as it provides improved pharmacokinetic properties including nucleolytic stability, bioavailability, and cellular uptake as a function of increased protein binding of the more lipophilic phosphorothioate (PS). In order to secure a very high stability towards enzymatic degradation and a high affinity towards the target mRNA (i.e. high Tm) the PS modification is routinely used together with high affinity carbohydrate modifications (i.e. high Tm) such as LNA, cET, and MOE. [10].

**Fig 1:**
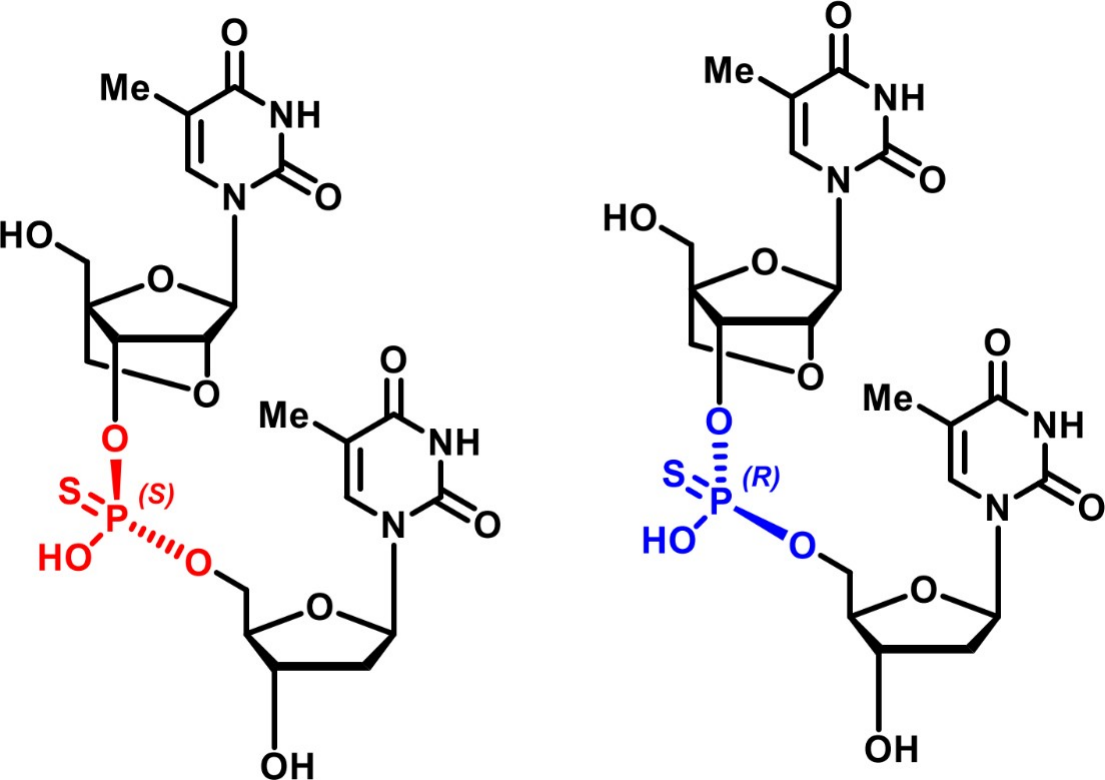
The two different diastereoisomers of a phosphorothioate LNA-T-DNA-T dimer.

However, with the introduction of a PS internucleoside linkage a chiral center is created at phosphorous (Rp or Sp, Fig 1). In the classic synthetic procedure for making PS LNA oligonucleotides (LNAs) the PS stereochemistry is not controlled and thus the LNAs actually consist of a large number of different diastereoisomers (*i*.*e*. 2^n^, n = internucleoside linkages). [11, 12] As an example, a 16-mer PS oligonucleotide, containing 15 internucleoside PS linkages, comprise 2^15^ = 32768 different diastereoisomers. Diastereoisomers are generally known to exhibit different chemical and physical properties, and therefore, diastereoisomers will also exhibit different pharmacological properties. To this end the drug properties of conventional oligonucleotide PS random mixtures are actually to be considered as the average biological response of a vast pool of structurally related compounds. Given that all possible stereochemical *R*/*S* combinations, in theory, are present within a conventional PS oligonucleotides the individual contributions of single PS centers are, in a random mixture, nearly negligible. [1] However, there will be diastereoisomers that differ significantly in properties compared with the average “pool” property of the random mixture. Therefore, the approach of making one or more stereodefined PS centers offers the opportunity to create isomeric sub-libraries or even fully stereodefined *single*-isomers with improved drug properties. [13]

Recently, we have published on the structural differences between fully stereodefined PS LNA oligonucleotides in a quantum mechanical computational study. [14, 15] A key finding was that the global structures and electrostatics of the LNAs are dependent on and controlled by the backbone chirality. As a result, even a few changes, down to a single PS-bond configuration, in the backbone can give rise to dramatic changes in the global structure and electrostatics which also may lead to changes in the drug properties.

Generally, PS stereochemistry has not been a major focus point during the last decade as the oligonucleotide optimization strategies were mainly focusing on other structural units, *i*.*e*. carbohydrate modifications. [1, 16, 17] These modifications in various combinations *e*.*g*. altering the ratio between DNA, LNA or other nucleotides have been widely studied. Thus, a focus on PS chirality adds a new perspective on the structure activity relationship (SAR) of therapeutic PS oligonucleotides in which the optimization is neither on the carbohydrate structure nor the nucleobase sequence.

For decades small molecule drug discovery has focused equally on controlling the stereochemistry as controlling any other structural unit. It is well established that stereochemistry is an essential driver for drug properties and the safest and most potent drugs are in most cases pure isomers. Since therapeutic oligonucleotides are acting in the same chiral biological environment isomerism ought to be equally important for RNA therapeutics. The therapeutic importance of PS chirality has recently led to a controversy in the literature giving rise to several conflicting opinions. [1, 18, 19, 20, 21] Iwamoto et al. and Hagedorn et al. showed that fully stereodefined PS oligonucleotides exhibited significant benefits in potency as compared to the “parent” diastereoisomeric random mixture. Hall et al., however, showed a limited benefit for PS isomeric sub-libraries on potency when only the flanks of MOE gapmers were stereodefined. In addition, a recent study by Østergaard et al. showed, for PS isomeric gapmer sub-libraries where only the DNA segment was stereodefined, and the cEt flanks were left undefined, neither an improvement in potency nor in safety.

Here, we have investigated the parameters that define the therapeutic index (*i*.*e*. safety and efficacy) by modulating PS chirality in a 16mer (*i*.*e*. 16mer is defined as an oligonucleotide having 16 nucleosides). We selected a 16mer random mixture PS LNA gapmer (C1) targeting the mRNA coding for the mouse myeloid differentiation primary response 88 (myD88) protein. This LNA gapmer was selected due to its unusual toxicity profile that was demonstrated in two recently established *in vitro* assays predictive of *in vivo* hepatotoxicity and nephrotoxicity. [22, 23] We show here that the safety profile of this LNA random mixture can be significantly manipulated by controlling the chirality of the internucleoside phosphorothioates. This adds important information to the ongoing discussions on the importance of stereodefined phosphorothioate oligonucleotides in RNA therapeutics.

## Materials and methods

All procedures were conducted in strict adherence to the Swiss federal ordinance on animal protection and welfare, according to the rules of the Association for Assessment and Accreditation of Laboratory Animal Care International (AAALAC), and with the explicit approval of the local veterinary authority (Kantonales Veterinäramt Basel-Stadt, Switzerland).

*In vitro* toxicity assessment is based on cellular systems that take up LNA oligonucleotides under gymnotic conditions, *i*.*e*. without any assisting formulation (gymnotic stems from Greek: gymnos = naked). [24] For the hepatotoxicity assessment LNAs were tested in primary mouse hepatocytes and cytotoxicity was determined by measuring increases in extracellular lactate dehydrogenase (LDH) and changes in intracellular ATP levels. [19] In the nephrotoxicity assay immortalized human renal primary proximal tubule epithelial cells (herein PTEC) were exposed to the LNAs in epidermal growth factor (EGF) containing medium followed by measurements of EGF consumption after 6 days. Elevated EGF levels in the supernatant indicate impairment of cellular integrity. [19] Both assays have been described previously. [18, 19] All details are furthermore provided in the S1 File at page 5.

The mRNA knock down was measured in the primary mouse hepatocyte assay only, together with the measurement of the cellular content of LNA.

When tested in primary mouse hepatocytes and human proximal tubular epithelial cells treatment with C1 resulted in “*severe toxicity”* in both cell systems.

## Results and discussion

We investigated if the toxicity of the compound could be altered by defining the stereochemistry of specific PS linkages along the backbone. For each phosphorous center kept in a specific stereo-configuration the number of diastereoisomers is reduced by a factor of two thus creating pools of compound sub-libraries from the “parent” random mixture.

**Fig 2:**
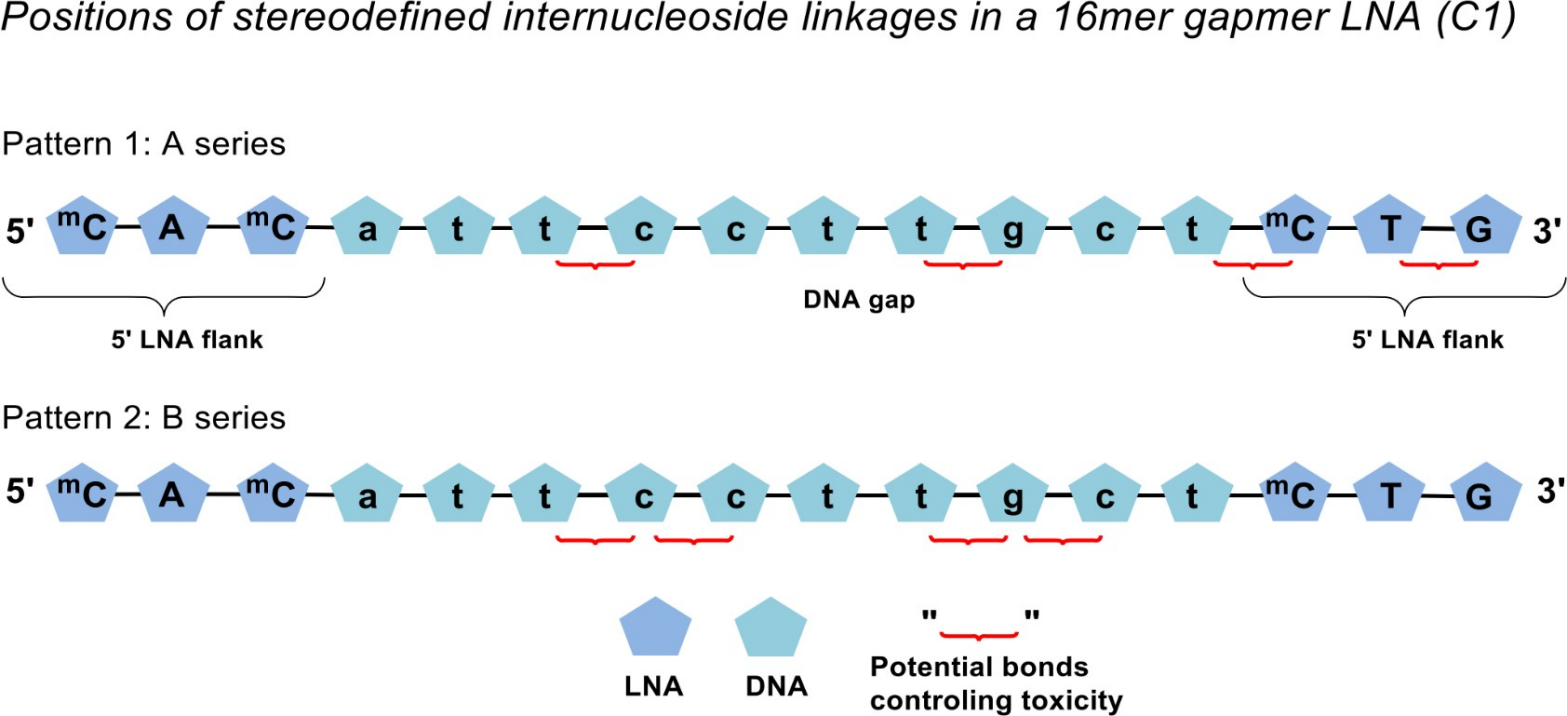
The 3-10-3 design of the oligonucleotide gapmer C1 targeting myD88 mRNA and the two stereodefined PS patterns with marked positions selected for stereodefinition.

We identified the positions where we wanted to control PS stereochemistry by using a previously published “LNA oligonucleotide hepatotox predictor”. [25] This algorithm was constructed by linking di-nucleotide motifs from a sub-set of antisense oligonucleotides which historically have been found to show hepatotoxicity *in vivo*. By using this algorithm it is possible to predict a potential hepatotoxicity with an ~80% probability for any given LNA oligonucleotide. The algorithm was used to define the di-nucleotide pairs shown in Fig 2 for which the internucleoside phosphates were selected for sites of stereodefinition.

Each pattern consists of four stereodefined internucleoside positions (red curly bracket Fig 2) while the remaining PS internucleoside linkages are synthesized as a random mixture of *R* and *S* configurations using conventional PS phosphoramidite chemistry. [26]

In the first pattern four separated di-nucleotide motifs were chosen, and in the second pattern the four di-nucleotide motifs were condensed into two groups (Fig 2). In each case the four selected internucleoside PSs were synthesized as all combinations of *R* or *S* stereoconfigurations, giving rise to 16 compound sub-libraries with all the other PSs not stereodefined. With the introduction of four stereodefined PSs the total amount of possible diastereoisomers was dramatically reduced from 32768, in the parent random mixture (C1), to only 2048 possible diastereoisomers in the new compound sub-libraries.

The 32 selected compound sub-libraries (16 compound sub-libraries for pattern 1 and 16 for pattern 2) were synthesized using a modified procedure from Wada et *al*., and were purified by standard HLB extraction reverse phase cartridge procedures (Table 1). [27, 28, 29, 30] The compound sub-libraries were characterized by mass spectrometry (see S1 File) and used in the biological assays as a formulation in PBS.

**Table 1:** Schematic representation of the compound sub-libraries.

Pattern 1		Pattern 2	
Oligonucleotide	Stereochemistry (5'-3')	Oligonucleotide	Stereochemistry (5'-3')
**A1**	xxxxxSxxxRxxRxSH	B1	xxxxxSRxxRSxxxxH
**A2**	xxxxxSxxxSxxRxRH	B2	xxxxxSSxxRRxxxxH
**A3**	xxxxxRxxxSxxSxRH	B3	xxxxxRSxxSRxxxxH
**A4**	xxxxxRxxxRxxSxRH	B4	xxxxxRRxxSRxxxxH
**A5**	xxxxxSxxxSxxRxSH	B5	xxxxxSSxxRSxxxxH
**A6**	xxxxxRxxxRxxSxSH	B6	xxxxxRRxxSSxxxxH
**A7**	xxxxxSxxxRxxRxRH	B7	xxxxxSRxxRRxxxxH
**A8**	xxxxxSxxxRxxSxSH	B8	xxxxxSRxxSSxxxxH
**A9**	xxxxxSxxxSxxSxSH	B9	xxxxxSSxxSSxxxxH
**A10**	xxxxxSxxxSxxSxRH	B10	xxxxxSSxxSRxxxxH
**A11**	xxxxxRxxxSxxSxSH	B11	xxxxxRSxxSSxxxxH
**A12**	xxxxxRxxxSxxRxRH	B12	xxxxxRSxxRRxxxxH
**A13**	xxxxxRxxxSxxRxSH	B13	xxxxxRSxxRSxxxxH
**A14**	xxxxxSxxxRxxSxRH	B14	xxxxxSRxxSRxxxxH
**A15**	xxxxxRxxxRxxRxRH	B15	xxxxxRRxxRRxxxxH
**A16**	xxxxxRxxxRxxRxSH	B16	xxxxxRRxxRSxxxxH

Schematic representation of the two sets of compound sub-libraries (pattern 1 and pattern 2) highlighting the positioning of R or S stereodefinition. The configurations are indicated by *R* or *S*, x nominates a non-defined phosphorothioate center and H marks the 3’-OH.

### *In vitro* toxicity profile of stereodefined LNA gapmers

All compounds described in Table 1 were initially screened for both hepatotoxicity and nephrotoxicity *in vitro*. The aim with the initial screen was to asses if the toxicity read-out was influenced by making partially stereodefined LNA gapmers (compound sub-libraries).

In both assays (data in S1 File from page 60) the overall toxicity profile of the compound sub-libraries was changed relative to the toxic parent random mixture. Thus, the safety profile of the PS oligonucleotides can be affected by a simple reduction of the number of possible diastereoisomers, which allows the design of a compound sub-library with lower toxicity.

From the initial screen 11 out of the 32 compound sub-libraries were chosen for further evaluation by also including measurements for target mRNA knock-down and cellular uptake (S1 File page 63).

**Fig 3:**
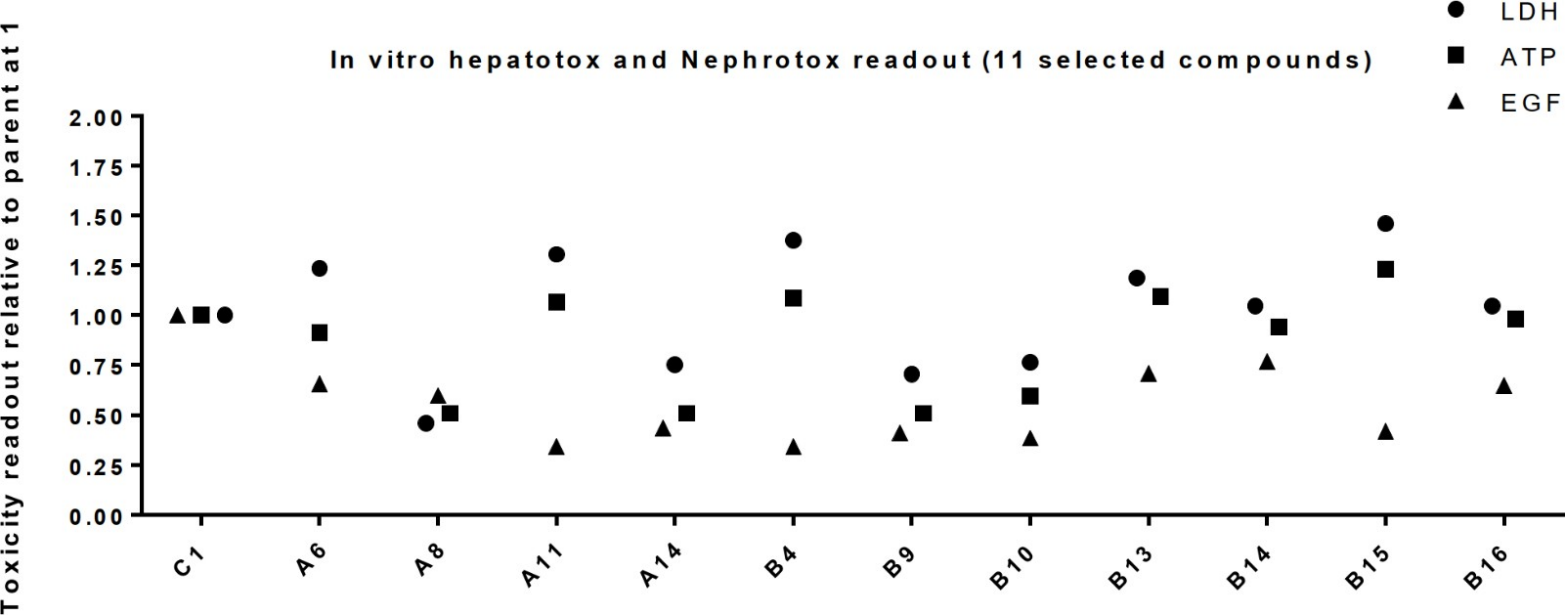
Differences in average *in vitro* toxicities for 11 selected compound sub-libraries (*n = 3*). Biomarkers for *in vitro* hepatotoxicity, LDH and ATP, were measured at 30 μM, and for nephrotoxicity, EGF, was measured at 100 μM. All averages of toxicity measurements were normalized to the parent random mixture and referenced to 1.

Fig 3 shows the changes of intracellular ATP and secreted LDH in primary mouse hepatocytes (hepatotoxicity assay), and the accumulation of EGF in the supernatant from human PTEC cells (nephrotoxicity assay) compared to the parent random mixture, C1. Again, significant toxicity changes were observed.

**Fig 4:**
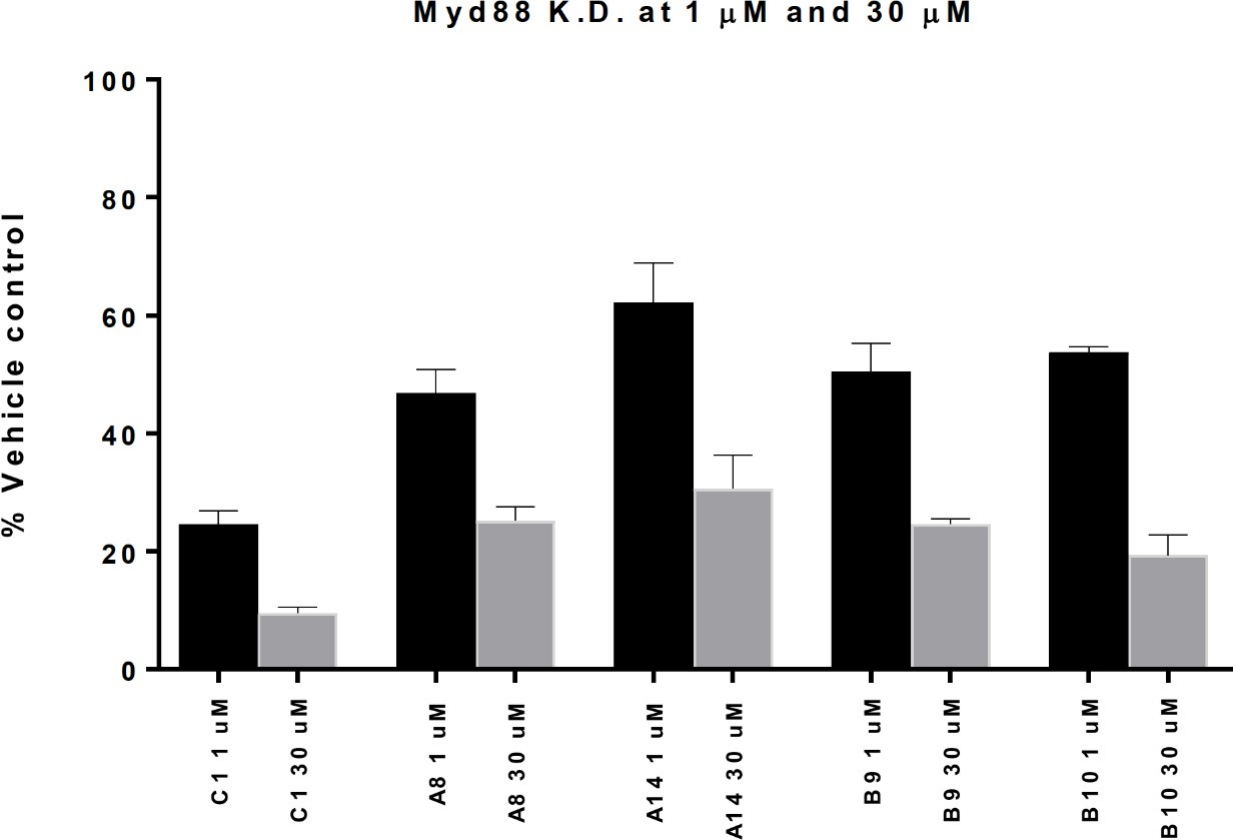
The target knockdown of the parent random mixture (C1) compared to 4 compound sub-libraries which have obtained an overall improved safety profile. All expression data were normalized to the house keeping gene RPS12. [32] Analyzing the changes in knock down at 30 μM and at 1 μM the values are observed to be significantly different at *n = 3* using the one-way ANOVA analysis with a significance level of p < 0.05 (calculated *p* value for both of 0.0001). In addition, all values at 1 μM and 30 μM are significantly different from the parent (C1) using the Dunnetts multiple comparisons test at p < 0.05.

Compound sub-libraries A8, A14, B9, and B10 scored 25 to 50% lower hepatotoxic values and approximately 50% lower nephrotoxic compared with the parent random mixture (Fig 3). These compound sub-libraries were also found to be less cytotoxic as determined by their ability to activate the apoptosis marker Caspase3/7 after transient transfection into HepG2 cells (see S1 File page 63). [31] However, in all four cases the potency measured by Myd88 knock down in mouse hepatocytes, compared to the parent random mixture, was also found to be lower at both 1 μM and 30 μM (Fig 4).

**Fig 5:**
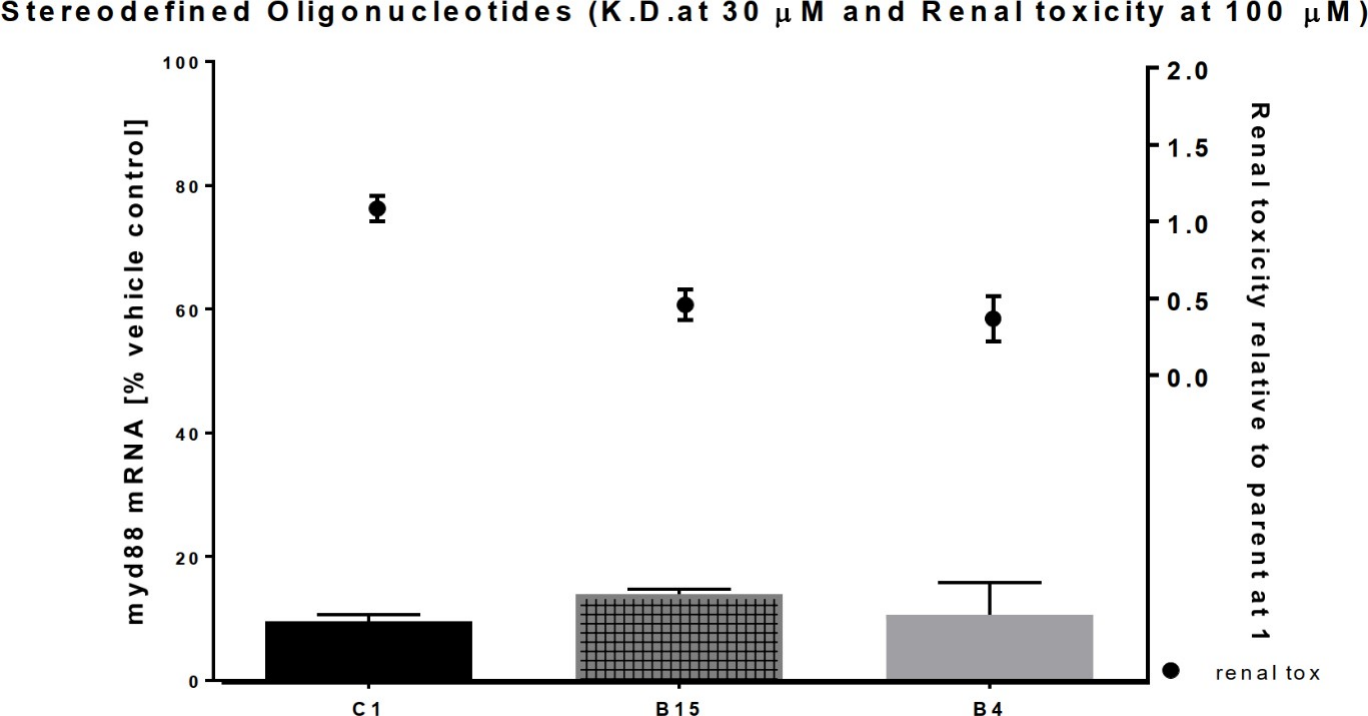
Two compound sub-libraries B4 and B15 of the toxic parent C1 are shown to retain target knock down in primary mouse hepatocyte while being approximately 25 to 50% reduction in toxicity in the nephrotoxicity assay (*n* = 3).

For many of the compound sub-libraries the on-target mRNA knockdown was reduced (up to a factor of approx. 3). However, some of the compound sub-libraries retained the potent target knockdown as the parent random mixture (Fig 5) while demonstrating an improved *in vitro* safety profile in the kidney cells. Therefore, by controlling the stereochemistry of just a few PSs along the backbone is one approach to identify *iso*-sequential compound sub-libraries with an improved therapeutic index.

It is interesting to note the diversity among the compound sub-libraries. For instance, compound sub-libraries B4 and B15 show no reduction in toxicity within the hepatotox assay (severe toxicity readout) as compared to the parent. However, when investigated in the nephrotoxicity assay the toxicity score was reduced more than 50% (severe toxicity to medium toxicity). Taking a closer look at the knock-down of the target mRNA (Fig 5) for both compound sub-libraries the kidney toxicity was reduced while retaining mRNA knock-down in primary mouse hepatocyte (% mRNA remaining vs. PBS control is: B4 at 11% +/-5 vs. B15 at 14% +/- 1 vs. parent at 10% +/-1).

**Fig 6:**
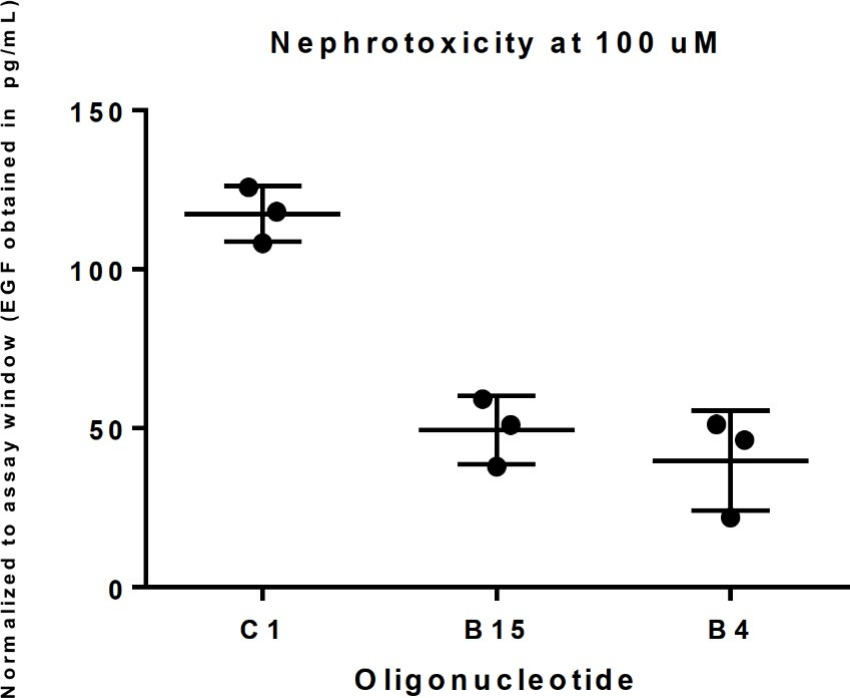
Nephrotoxicity score for two compound sub-libraries (B15, B4) with same potency as the parent random mixture (C1) at 100 μM (*n* = 3).

Using the one-way analysis of variance (ANOVA) there is no statistically significant differences (p > 0.28) between the average mRNA knock down of the parent and the two compound sub-libraries while the EGF readouts were statistically significant (Fig 6).

Further analyzing the changes in nephrotoxicity at 100 μM the findings are seen to be statistically significant at *n = 3* using the one-way ANOVA analysis with a significance level of p < 0.05 (calculated *p* value of 0.0004) (Fig 6). While mRNA knock-down in hepatocytes and hepatotoxicity at 30 μM is retained, the nephrotoxicity safety profile of the compound sub-libraries has been significantly improved.

In order to investigate whether *in vitro* toxicity levels in liver and kidney cells is related to potency in the target cells (hepatocytes) the two parameters were plotted against each other and the Pearson correlation was calculated (see S1 File from page 65). It was seen that the correlation between toxicity and mRNA knock-down gave R^2^ values of 0.2 and a Pearson correlation analysis of -0.44 for the relationship between renal toxicity and mRNA knock down. For LDH correlated to mRNA knock down an R^2^ of 0.5 with a Pearson correlation analysis of -0.70 was obtained and for ATP correlated to mRNA knock down an R^2^ of 0.7 with a Pearson correlation analysis of -0.82 was obtained. The data of correlation is thus not conclusive. It is, however, possible to find a specific stereochemistry along the backbone which can control a single toxicity parameter selectively and leave the target knock-down unaffected.

**Fig 7:**
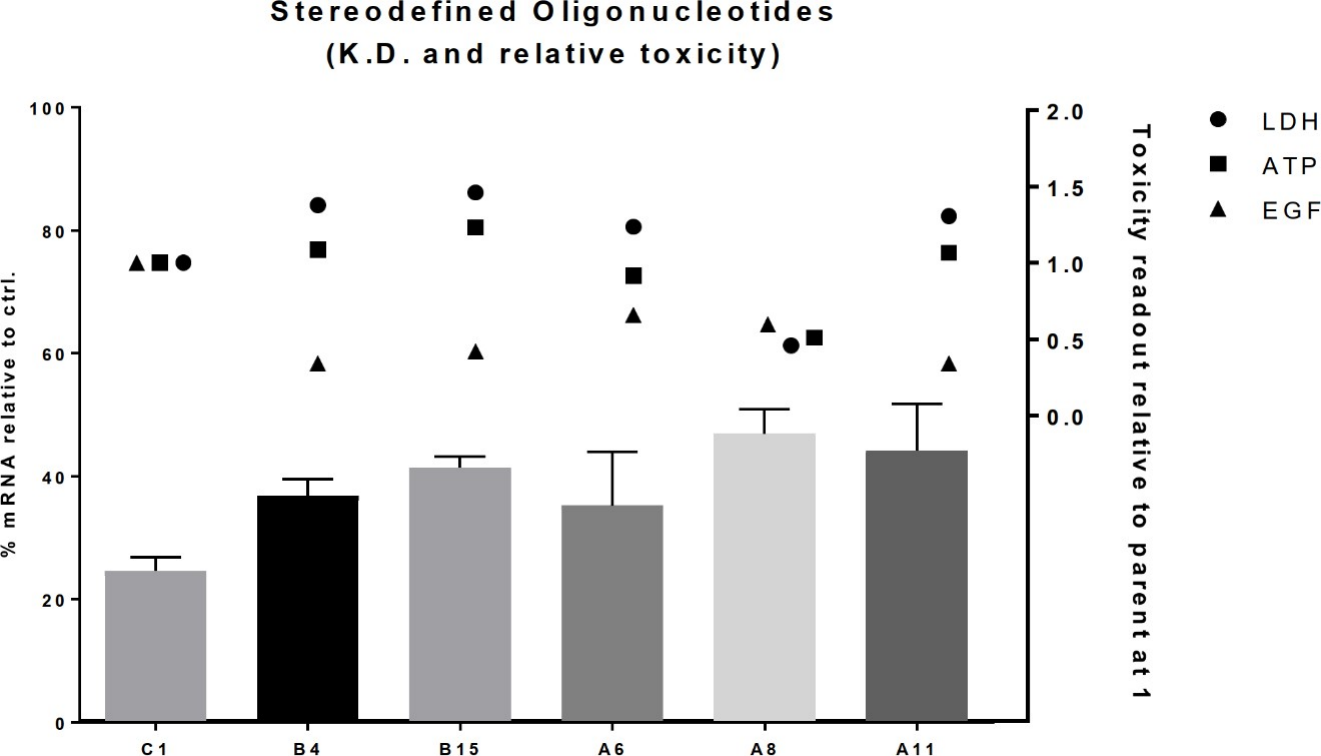
Knock-down at 1 μM and average relative toxicity levels of five compound sub-libraries and the parent random mixture C1 included as reference.

We further compared toxicity and knock-down for five compound sub-libraries (B15, A6, A8, A11, and B4) with the parent random mixture (C1) (Fig 7). For all five compound sub-libraries a statistical significant difference in LDH, ATP, or EGF levels compared to C1 was detected, while the average mRNA knock down of myd88 in mouse hepatocytes was shown not to be significantly different (ANOVA analysis with calculated *p* = 0.14 at *p* < 0.05). This finding thus points towards that the observed toxicity can be unrelated to the level of mRNA knock down induced by the oligonucleotide.

Finally, we investigated the cellular uptake (*i*.*e*. intracellular concentration) of the compound sub-libraries. Uptake was measured in mouse hepatocytes in parallel to knock-down and toxicity assessments. It was observed that the uptake of the compound sub-libraries varied with a factor of up to 1.5 compared to the parent random mixture (page 63 S1 File). The changes in cell-uptake at 30 μM and at 1 μM are statistically significant at *n = 3* using the one-way ANOVA analysis with a significance level of p < 0.05 (calculated *p* value of 0.0004 and 0.0001 respectively). At 30 uM, except for C1 vs A11 and C1 vs B4, the changes are all significant using Dunnetts multiple comparisons test at p < 0.05. In addition, all measurements are significantly different from C1 at 1 uM (Dunnetts multiple comparisons test at p < 0.05). Although the exposure is not markedly different it shows that backbone stereochemistry can also influence cellular uptake but since the difference in uptake observed here is rather small we do not assign the differences in the observed *in vitro* toxicity to this attribute (see data in S1 File at page 63).

### Structure activity relationship

In order to further understand the structure activity relationship (SAR) a closer analysis of the backbone stereochemistry was carried out. Compound sub-libraries B9 and A8 with pattern 1, and A14 and B10 with pattern 2, which all showed an improved *in vitro* safety profile in both the hepatotox and nephrotoxicity assay, shows distinct configurational patterns.

In pattern 1 (A8 and A14) the three stereodefined PS positions, from the 5’ end, are identical (xxxxxSxxxRxxSx**S**H and xxxxxSxxxRxxSx**R**H). The last position (bold) can in the two cases be either *R* or *S*, implying that this position might not be determinant controlling toxicity. In other words, either R or S in the ultimate 3’ position, coupled with configurational retention in the three other positions, could lead to the identification of “smaller” compound sub-libraries or even fully stereodefined *single* diastereoisomers exhibiting a further improved tolerance.

Pattern 2, which includes the compound sub-libraries B9 and B10 with *reduced* toxicity, also shares 3 out of 4 defined PS stereocenters (xxxxxSSxxS**S**xxxxH and xxxxxSSxxS**R**xxxxH). The bold position can in this case again be either *R* or *S* indicating that it is the other three positions that are determinant for toxicity, and as mentioned above, may lead the way to find even better tolerated diastereoisomers.

The compound sub-libraries B15 (xxxxxRRxxRRxxxxH) and B4 (xxxxxRRxxSRxxxxH) had reduced nephrotoxicity but exhibited unchanged mRNA knock-down and hepatotoxicity. This shows that in this case the R configuration for the backbone in pattern 2 is beneficial. In both cases 3 out of 4 positions are R (*i*.*e*. xxxxxRRxx**R**RxxxxH and xxxxxRRxx**S**RxxxxH). It seems here that position 10 (bold), from the 5’ end, can be either R or S without changing the measured properties. Collectively these results illustrate that some PS chirality positions are more determinant for therapeutic relevant properties. In addition, the analysis showcases that rules are not yet possible to define and generalize. However, as seen in the above, a sub-library approach where *e*.*g*. 4 out of 15 stereocenters are defined can give meaningful differences. Thus, in these cases, it is recommended to screen all possibilities (*i*.*e*. 16 different molecules for each pattern) and by this determine the stereochemical pattern that gives the optimal drug properties.

## Conclusion

In this study it was for the first time investigated whether the *in vitro* therapeutic index of a toxic phosphorothioate LNA gapmer could be changed by controlling PS chirality. The results demonstrate that the toxicity profile in the case of hepatotoxicity and nephrotoxicity can be manipulated as a function of the specific positioning of *R* and *S* stereochemistry along the backbone. We show that the toxicity readout for hepatotoxicity (*from estimated severe toxicity to medium toxicity*) and for nephrotoxicity (*from severe toxicity to medium toxicity*) could be modulated. In most cases a small reduction in target knock-down was also observed. It was, however, even for this small selection of compound sub-libraries, possible to find examples where the antisense activity was maintained, as compared to the parent (C1), whilst the nephrotoxicity signal was reduced by approximately 50%.

The experiments in this study have focused on a small section of compound sub-libraries of a 16mer LNA gapmer. Considering that the parent LNA random mixture was unusual toxic it is interesting to note that a significant reduction in toxicity can be observed by only fixing 4 stereo centers. Thus for oligonucleotides exhibiting low toxicity it might be possible to completely eliminate any toxicity score by selecting appropriate compound sub-libraries. In addition, by defining additional PSs in a sub-library, exhibiting e.g. a medium tox score, could potentially lead to a fully PS stereodefined single isomer and a further overall improved drug profile.

Gapmer oligonucleotide hepatotoxicity in mice has in some cases been reported to be associated with high target affinity. [33]To a great extent the observed toxicities were associated with off target effects on the transcriptome. We show here that the toxicity profile of a specific LNA gapmer is significantly reduced for selected compound sub-libraries that share the same nucleoside composition and nucleobase sequence as the parent random mixture only differing in their PS stereoconfiguration. Thus, the use of stereodefined internucleoside linkages can be used to manipulate oligonucleotide toxicity, thereby providing a useful tool in optimizing oligonucleotides for therapeutic use.

We conclude that stereo definition offers the opportunity for bespoke tailoring of antisense properties for PS oligonucleotides. The configurational rules underlying these mechanisms are not known at this point in time. Until the design rules are fully understood diligent screening is needed in order to identify compound libraries, or even single isomers, with improved properties.

## Supporting information

S1 File (DOCX)

## Abbreviations

LNA: Locked Nucleic Acid
PS: Phosphorothioate
LDH: lactate dehydrogenase
PTEC: proximal tubule epithelial cells
EGF: epidermal growth factor
