# Supplementary material for: Refining LNA safety profile by controlling phosphorothioate stereochemistry
Source: PLoS One. 2020 Jun 12;15(6):e0232603. doi: 10.1371/journal.pone.0232603 (PMC7292364; doi:10.1371/journal.pone.0232603)
Supplement: S1 File — (DOCX) [file pone.0232603.s001.docx]

**Table of contents**

Oligonucleotide synthesis experimentals P2

Methods for primary mouse hepatocytes P5

Methods and materials Nephrotoxicity assay P8

Spectra (UV 260 nm and MS ES-) for all 32 compounds (pattern 1 and pattern 2) P10

Summary table for pattern 1 and pattern 2 (UV purity, calculated mass, and found mass) P44

Spectra (UV and MS) for all 11 resynthesized compounds (pattern 1 and pattern 2) P45

Summary table for 11 resynthesized compounds UV purity, calculated mass, and found mass) P58

In vitro safety of initial 32 compounds P59

Cellular uptake, in vitro efficacy and in vitro toxicity of stereodefined LNA sub-libraries P61

Nephrotoxicity and hepatotoxicity as a function of mRNA knock down P63

Renal toxicity correlated to mRNA K.D. in mouse hepatocytes P64

ATP correlated to mRNA K.D. P65

LDH correlated to mRNA K.D. P66

Elaborated in vitro toxicity for figure 7 P67

Statistical methods used P71

**Oligonucleotide synthesis experimentals**

Stereodefined Amidites:

Stereodefined amidites D-DNA T, L-DNA T, D-DNA C, L-DNA C, D-DNA G, L-DNA G, D-LNA T, L-LNA T were obtained by the procedure of Wada et. *al* as seen in Oka, W.; Yamamoto, M.; Sato, T.; Wada, T. *J. Am. Chem. Soc.*, **2008**, *130*, 16031-16037.

Amidite solutions: Stereodefined amidites were dissolved at 0.1 M in 3.5% pyridine in MeCN, std. DNA amidites and std. LNA amidites were dissolved at 0.1 M in MeCN.

*General reagents used*

Solid support: 1 umol unylinker CPG

Detritylation: 3% Dichloroacetic acid in dichloromethane.

Activator: 1 M DCI, 0.1 M NMI in MeCN

Sulfurization: 0.1 M Xanthane Hydride in 1:1 pyridine and acetonitrile

Capping (Ac_2_O, pyridine, N-methylimidazole in MeCN) also known as Cap A and Cap B mixed in 1:1)

Removal of cyanoethyl (DEA wash): 20% Diethylamine in MeCN.

Deprotection from solid support and global deprotection: conc aq. NH_4_OH at 55 °C for 24 hours.

Synthesis cycle on 1 µmol scale adapted from:
Wan, W. B.; Migawa, M. T.; Vasquez, G.; Murray, H. M.; Nichols, J. G.; Gaus, H.; Berdeja, A.; Lee, S.; Hart, C. E.; Lima, W.F.; Swayze, E. E.; Seth, P. P. *Nucleic Acids Research*, **2014**, *42*, 13456-13468

1. Deprotection of solid support using 3% Dichloroacetic acid in dichloromethane.
2. MeCN wash
3. Coupling using activator and phosphoramidte (1 M DCI, 0.1 M NMI and amidites at 0.1 M in either MeCN (std. amidites) or 3.5% pyridine in MeCN (stereodefined amidites) – (3 x 3 min)
4. MeCN Wash
5. Sulfurization using 0.1 M Xanthane Hydride in 1:1 pyridine and acetonitrile
6. Capping (Ac_2_O, Lutidine, pyridine in MeCN)
7. Deprotection of DMTr group using 3% Dichloroacetic acid in dichloromethane
8. MeCN wash
9. Repeat steps 2-8 for next incorporation (stop at step 6 to finish with DMT-ON product)

After the elongation cycle was finalized the final product was released from the solid support and globally deprotected using conc aq. NH_4_OH at 55 °C for a minimum 24 hours. The product was purified as the DMTr-ON oligo using std. reverse phase cartridge purification.

**Methods for primary mouse hepatocytes**

**Mouse liver perfusion and hepatocyte isolation**

All procedures were conducted in strict adherence to the Swiss federal ordinance on animal protection and welfare, according to the rules of the Association for Assessment and Accreditation of Laboratory Animal Care International (AAALAC), and with the explicit approval of the local veterinary authority (Kantonales Veterinäramt Basel-Stadt, Switzerland).

Primary mouse hepatocytes were isolated from 10- to 13-week old male C57Bl6 mice by a retrograde two-step collagenase liver perfusion as described in **Sewing et al. (2016) PLoS ONE 11(7): e0159431. doi:10.1371/journal.pone.0159431**. Freshly isolated primary mouse hepatocytes were suspended in Williams medium E. (WME) supplemented with 10% fetal calf serum, penicillin (100 U/ml), streptomycin (0.1 mg/ml) at a density of approx. 5 x 10^6^ cells/ml and seeded into collagen-coated 96-well plates (Becton Dickinson AG, Allschwil, Switzerland) at a density of 0.25 x 10^5^ cells/well. Cells were pre-cultured for 4h allowing for attachment to cell culture plates before start of treatment with oligonucleotides. Seeding medium was replaced by 90 µl of serum free WME and 10 µl of oligonucleotide stock solutions in PBS were added to the cell culture and left on the cells for 3 days.

## LDH and ATP assays

Lactate dehydrogenase (LDH) released into the culture media was determined using a Cytotoxicity Detection Kit (Roche 11644793001, Roche Diagnostics GmbH Roche Applied Science Mannheim, Germany) according to the manufacturer's protocol. Intracellular GSH levels were determined in intact cells by a fluorescent assay using monochlorobimane (Fluka, 69899). For the determination of cellular ATP levels the CellTiter-Glo® Luminescent Cell Viability Assay (G9242, Promega Corporation, Madison WI, USA) was used according to the manufacturer's protocol. All Experiments were performed in triplicates

**Quantification of intracellular AON content by hybridyzation ELISA**

Intracellular LNA content was determined by hybridization dependent enzyme-linked immunosorbent assay (hELISA) using a biotinylated capture probe and a digoxigenin conjugated detection probe as described by **Straarup et al. (2010) Nucleic Acids Res. 2010;38(20):7100-11**. Mouse heptocytes were seeded and treated with AON as described above. After 2 days of incubation cells were washed with 0.1mg/mL heparin solution (Sigma H3393-25KU) and lysed in 75 µl of RLT Plus buffer (Qiagen 1053393). Cell lysates were diluted and incubated with 35 nM biotinylated capture probe and 30 nM Digoxigenin coupled detection probe for 30 min at room temperature in 5x Saline sodium Citrate buffer (20x SSCT, Sigma 6639) containing 0.05% Tween-30 (Sigma P9416) in a 96 well plate. The assembled complex is then captured on a streptavidine-coated ELISA plate (Nunc 436014) for 1 h and after three washing steps with 2xSSCT buffer, each well is incubated with an anti - Digoxigenin- Alkaline Phosphatase(AP)-Fab fragment (Roche 11093274910) for 1 h at room temperature. After three additional washing steps, Blue Phos Substrate (KPL 50-88-00) was added to the plates and color development was measured spectrophotometrically at 615 nm after 20 min. Concentration of LNA in the lysate was calculated according to a standard curve generated with the respective LNA stock solution.

**Caspase3/7 Activation**

Caspase-3/7 activity was determined using the Caspase-Glo® 3/7 Assay (Promega Corporation, Madison WI, USA). In brief, Caspase-Glo® 3/7 reagent was added to the cells at indicated time points, incubated for 30 min, before luminescence was determined on an Enspire multi-mode plate reader (Perkin Elmer) according to the manufacturer's instructions.

## RNA isolation and qPCR

mRNA purification from mouse hepatocytes was performed using the RNeasy 96 Kit (Qiagen, Hombrechtikon, Switzerland) including an RNAse free DNAse I treatment according to the manufacturer’s instructions. cDNA was synthesized using iScript single strand cDNA Synthesis Kit (Bio-Rad Laboratories AG, Reinach, Switzerland). Quantitative real-time PCR assays (qRT-PCR) were performed using the Roche SYBR Green I PCR Kit and the Light Cycler 480 (Roche Diagnostics, Rotkreuz, Switzerland) with specific DNA primers. Analysis was done by the ΔΔCt threshold method to determine expression relative to RPS12 mRNA. Each analysis reaction was performed in duplicate, with two samples per condition.

**Methods for Nephrotoxicity assay**

**Human PTEC culture**

Primary PTEC (Science cell 4100) and PTEC-TERT1 (Evercyte GmbH, Austria) were cultured according to the manufacturer’s instructions in PTEC medium [DMEM/F12 without phenol red (ThermoFisher Scientific 11039021) containing 1% Penicillin-Streptomycin solution (ThermoFisher Scientific 15140122), 10 mM HEPES (ThermoFisher Scientific 15630056), 5 µg/ml insulin and 5 µg/ml transferrin and 8.65 ng/ml sodium selenite (all from a 100x concentrated stock solution, ThermoFisher Scientific 41400045), 100 nM hydrocortisone (Sigma H6909), 3.5 µg/ml ascorbic acid (Sigma A4403), 25 ng/ml prostaglandin E1 (Sigma P5516), 3.25 pg/ml triiodo-L-thyronine (Sigma T6397), 10 ng/ml human recombinant epidermal growth factor (EGF, R&D Systems 236-EG-200), and 100 µg/ml Geneticin (G418 sulfate, ThermoFisher Scientific 10131027)].

**For AON toxicity assessment**, PTEC-TERT1 were seeded into collagen I-coated 96-well plates (Corning, 356407) at a density of 20 000 cells/well respectively in PTEC medium and grown until confluence prior to treatment with AONs. AONs were dissolved in PBS and added to the cell culture at a final concentration of 30 or 100 µM in a final volume of 100 µl. Medium was changed, stored at -20ᵒC for cytokine analysis and refreshed along with AONs after 3 days. PBS served as vehicle control. For analysis of human EGF at day 6, cell supernatants were thawed on ice, diluted 1:2 and 1:10 in sample dilution buffer (BioRad catalog # M60-009RDPD) and analyzed by Luminex-based ELISA using human EGF beads (Bio-Plex Pro™ Human Cancer Biomarker Panel 2 EGF Set#171BC603M), followed by analysis using the Bio-Plex® 200 Systems (BioRad) according to the manufacturer’s instructions. Data are reported as mean concentrations and standard deviations of triplicate wells.

**Spectra (UV 260 nm and MS ES-) for all 32 compounds (pattern 1 and pattern 2) + blank run (Milli-Q® water injected)**

**Representative sample of background: “blank run” using 10 µL Milli-Q® water as the injected sample.**


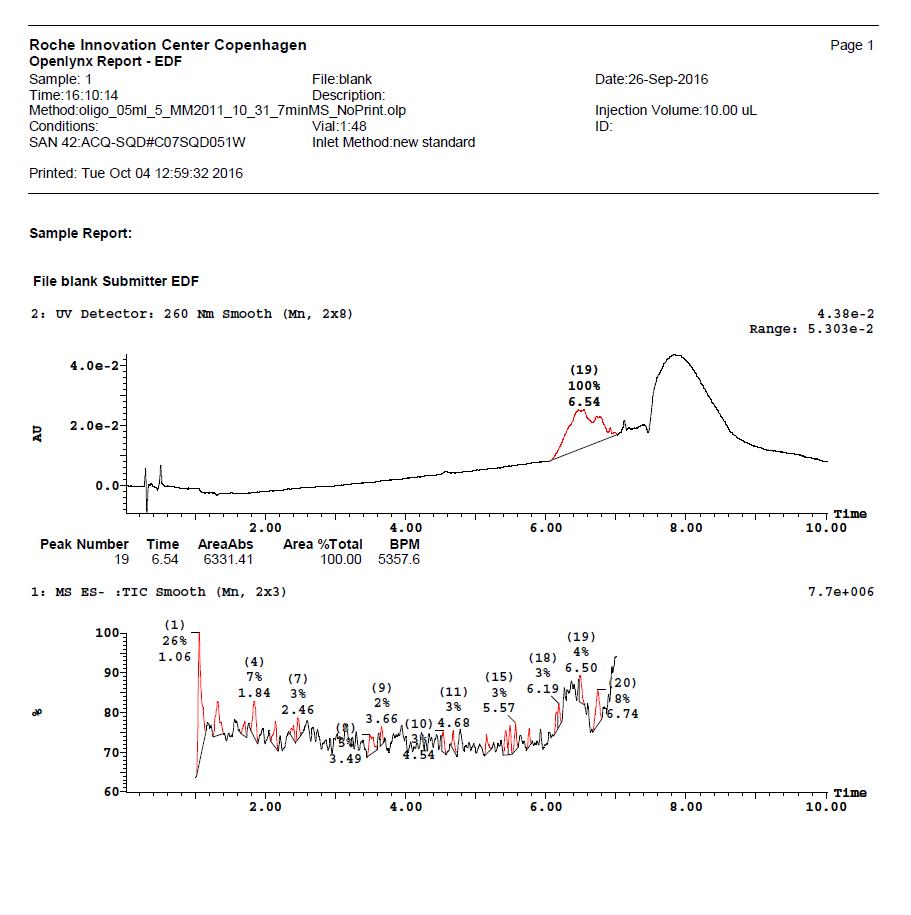


A1 (Tox1)


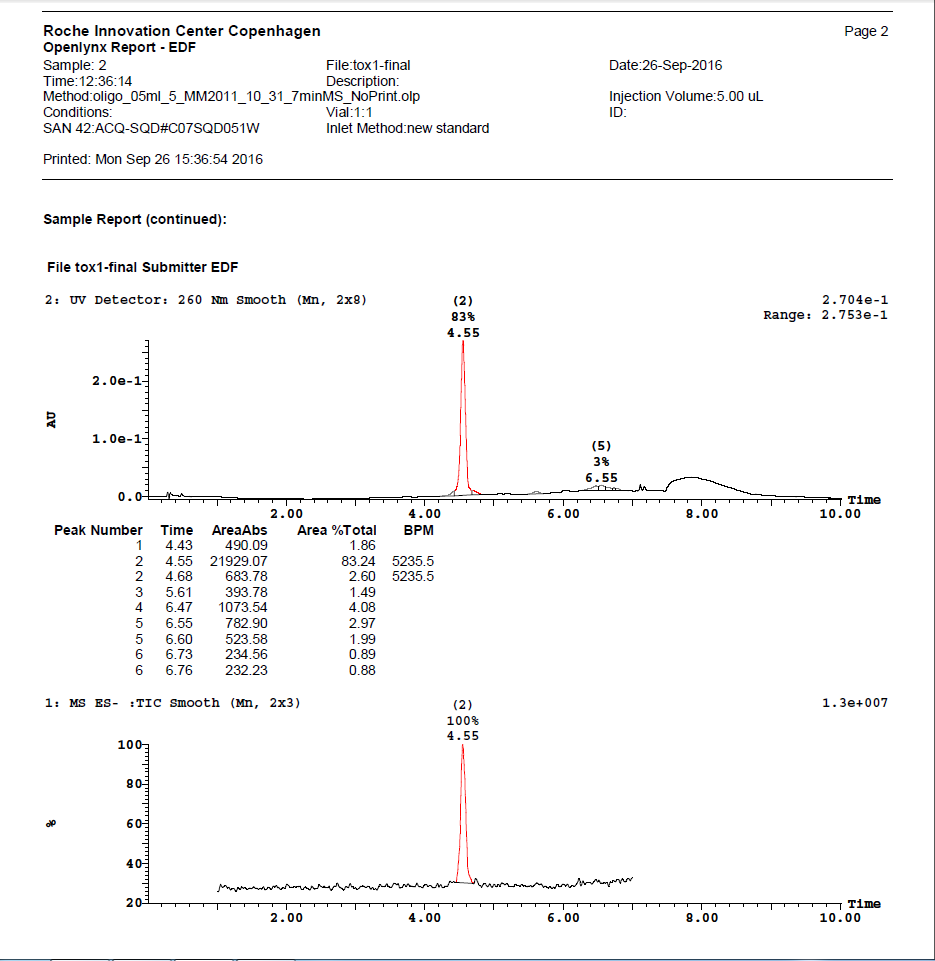


A2 (Tox2)


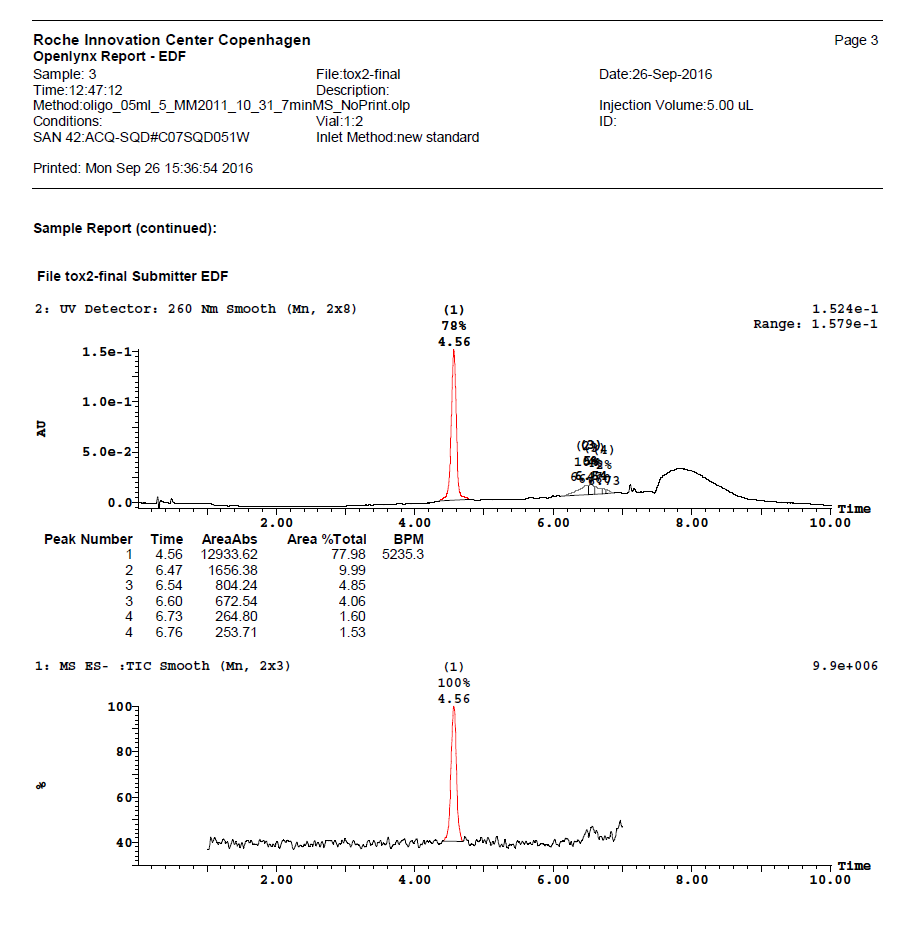


A3 (Tox3)


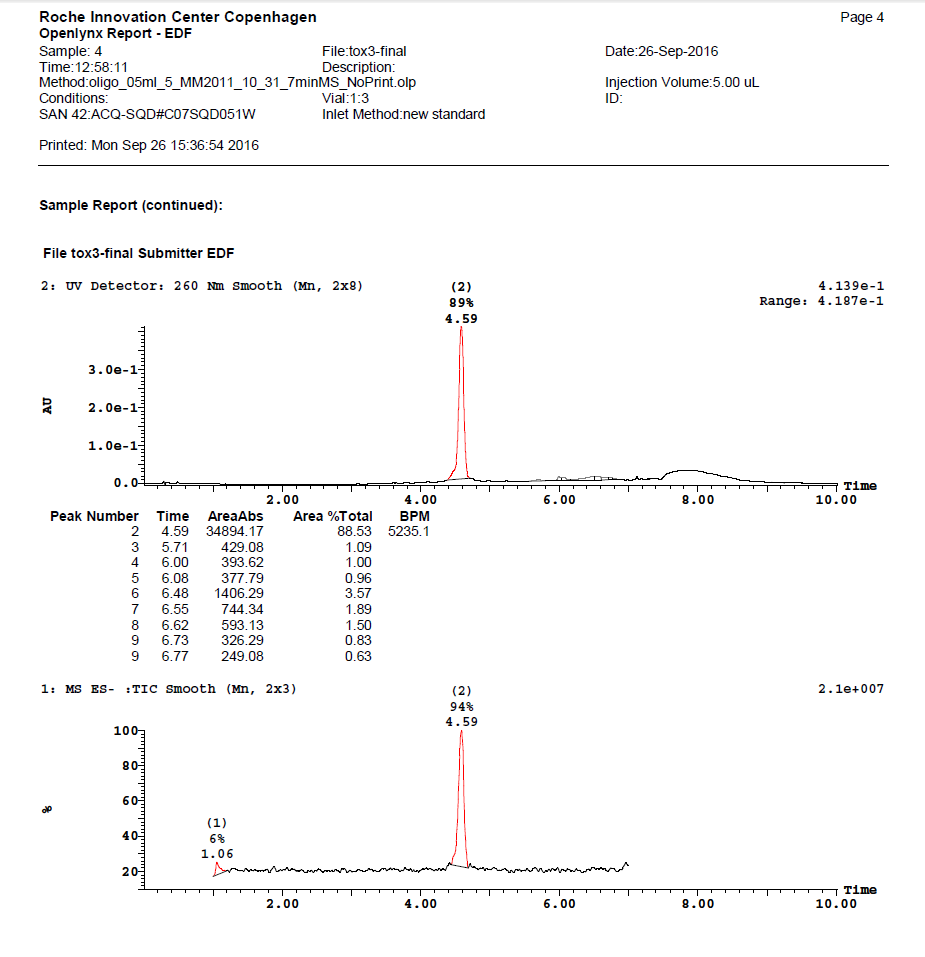


A4 (Tox4)


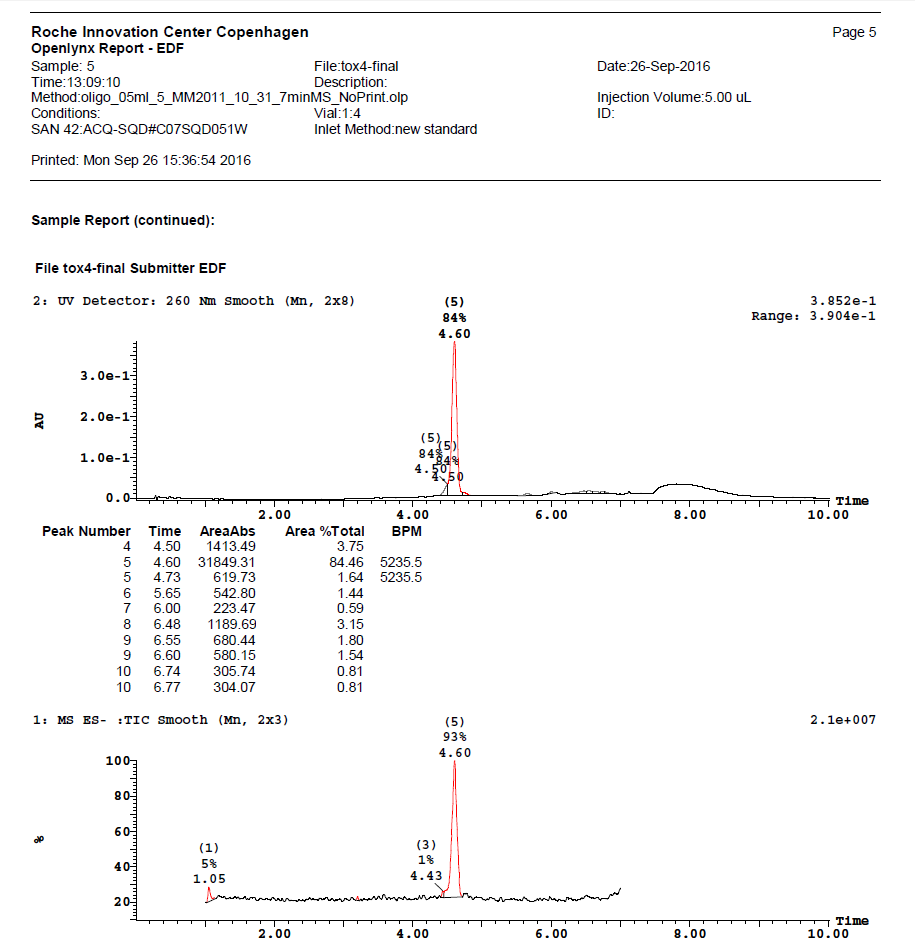


A5 (Tox5)


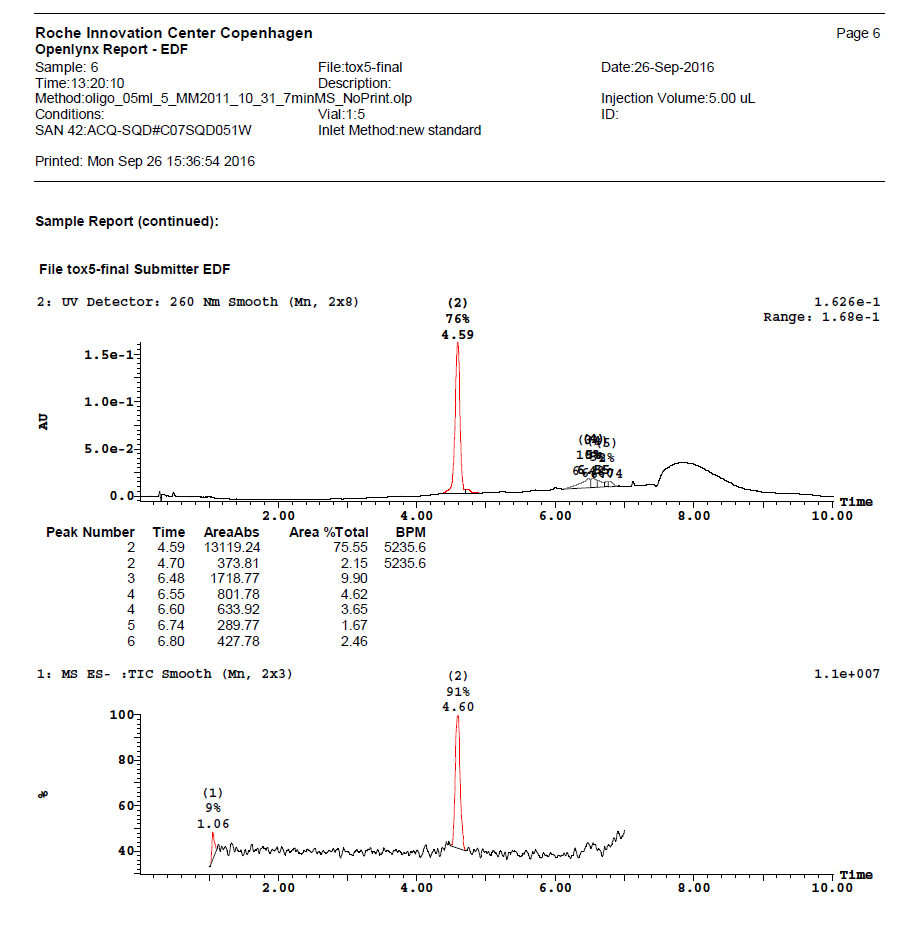


A6 (Tox6)


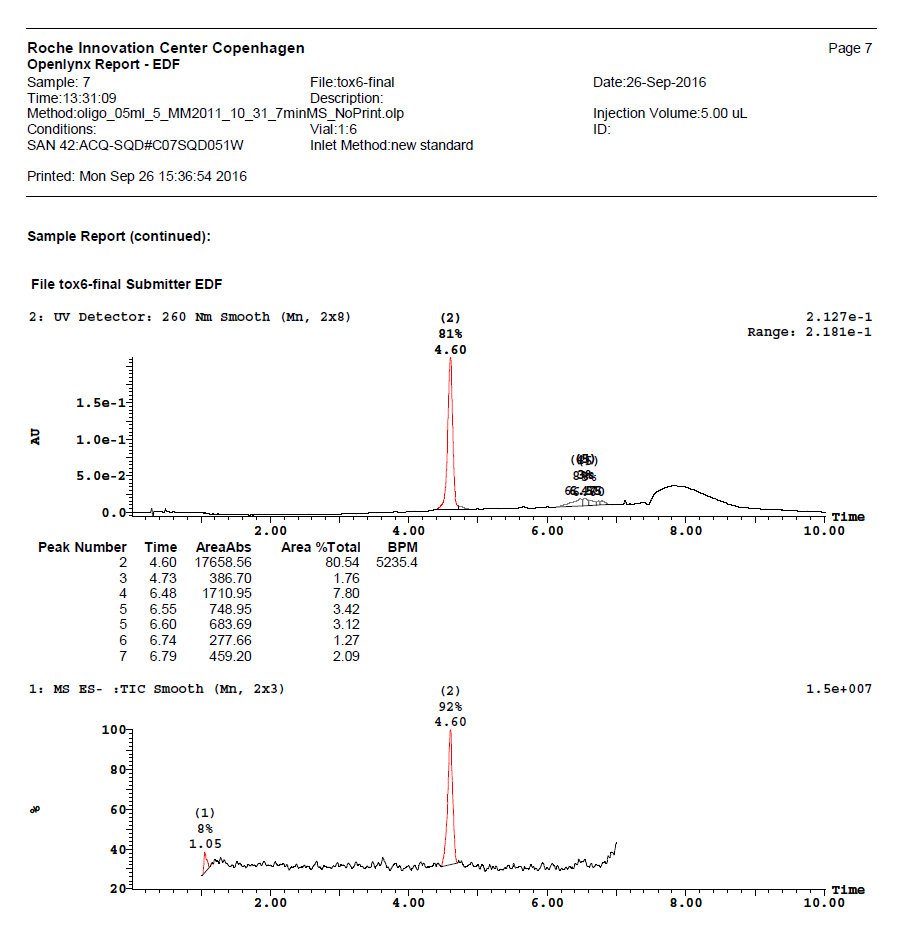


A7 (Tox7)


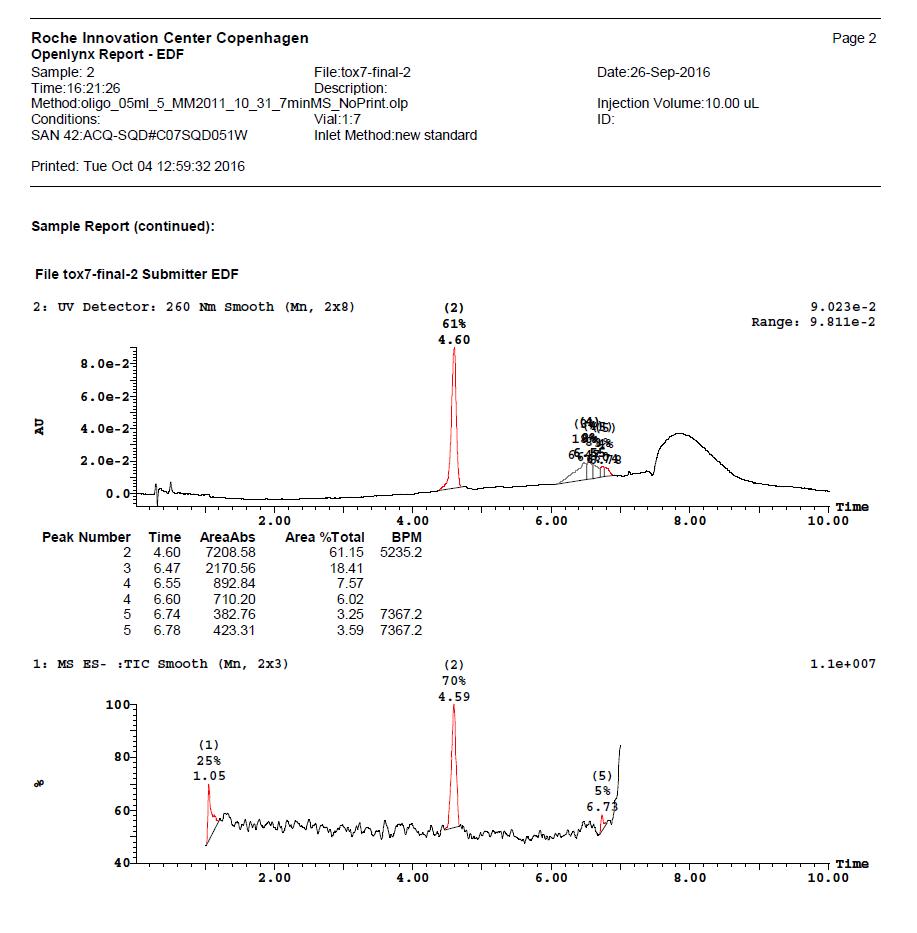


A8 (Tox8)


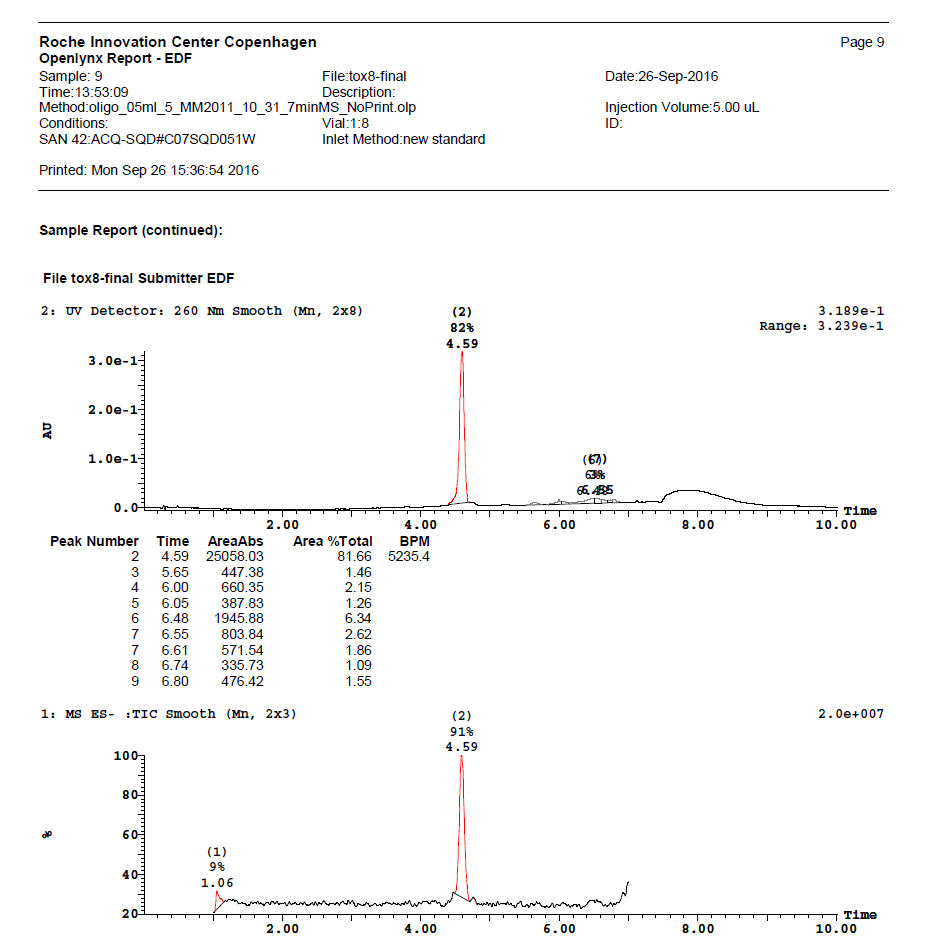


A9 (Tox9)


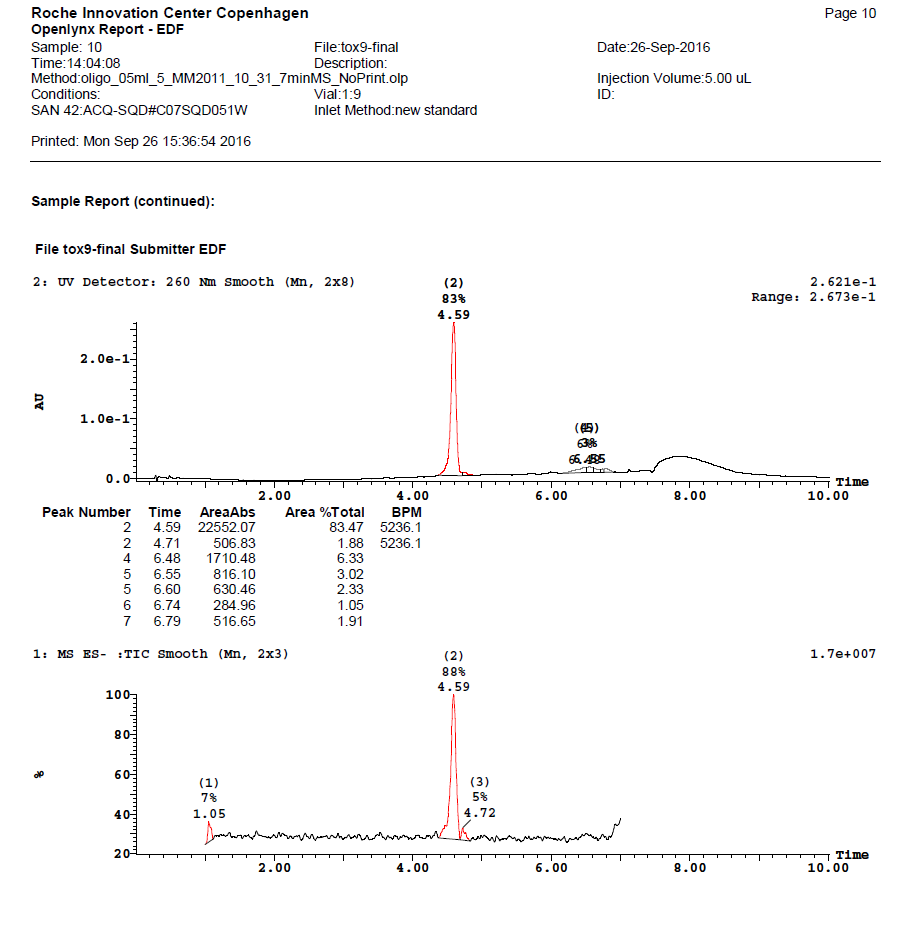


A10 (Tox10)


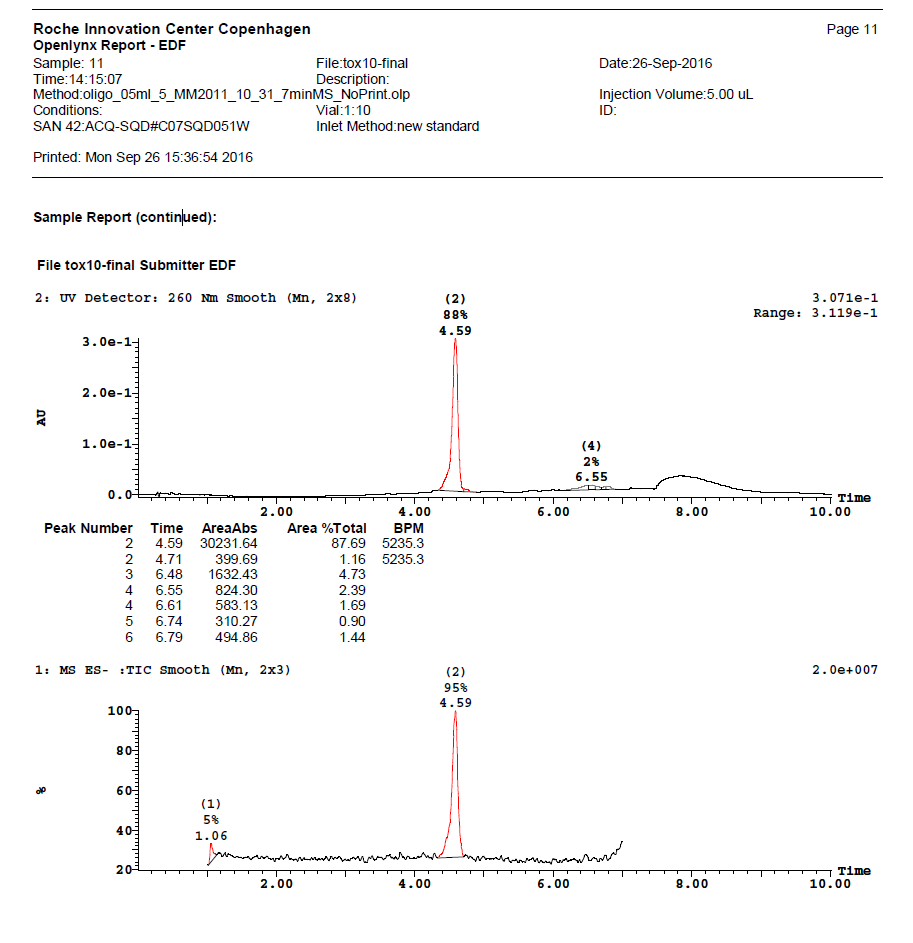


A11 (Tox11)


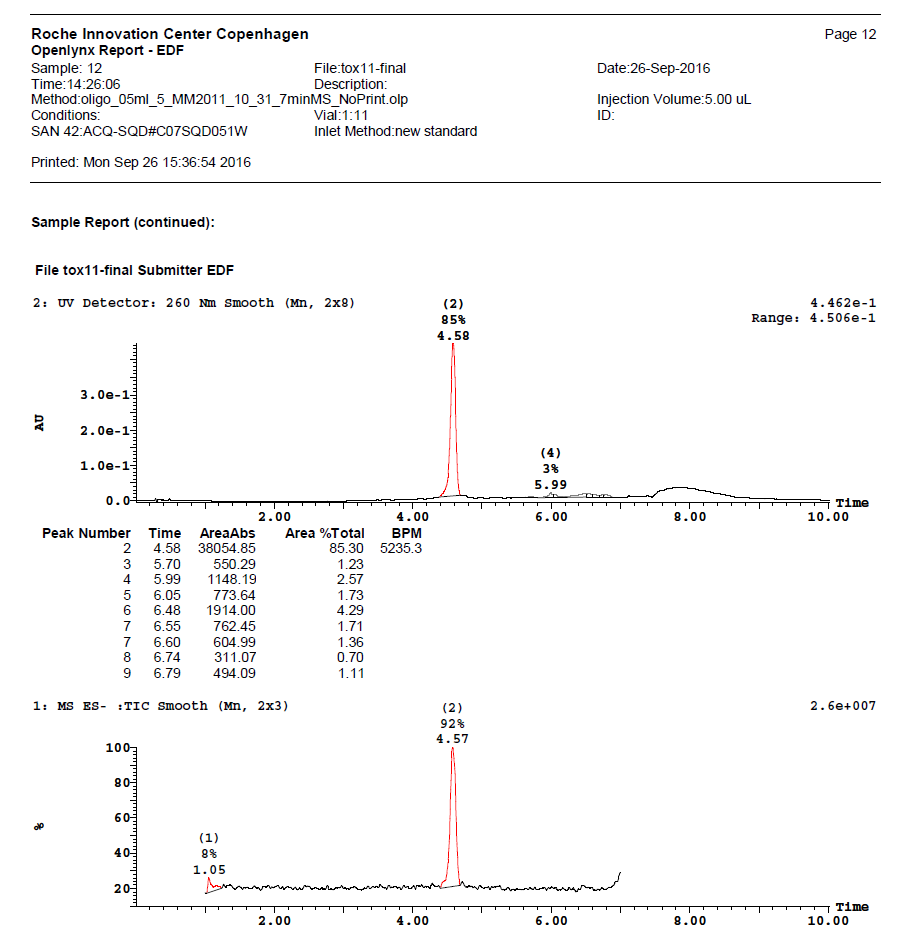


A12 (Tox12)


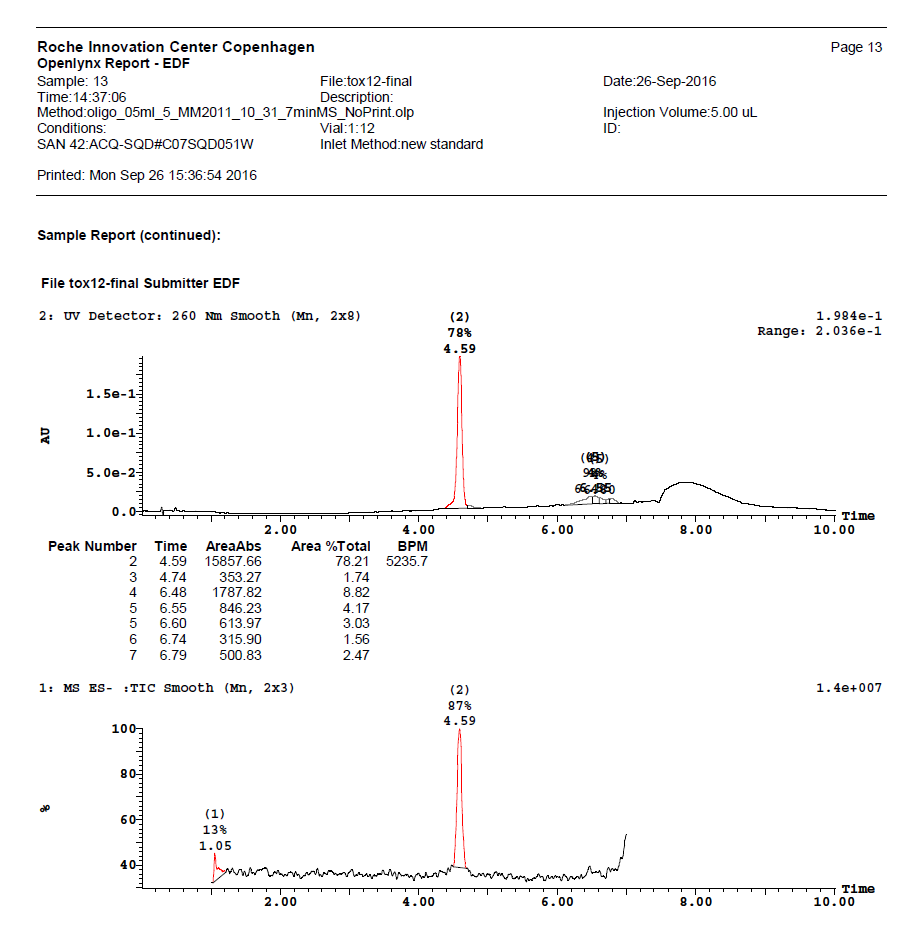


A13 (Tox13)


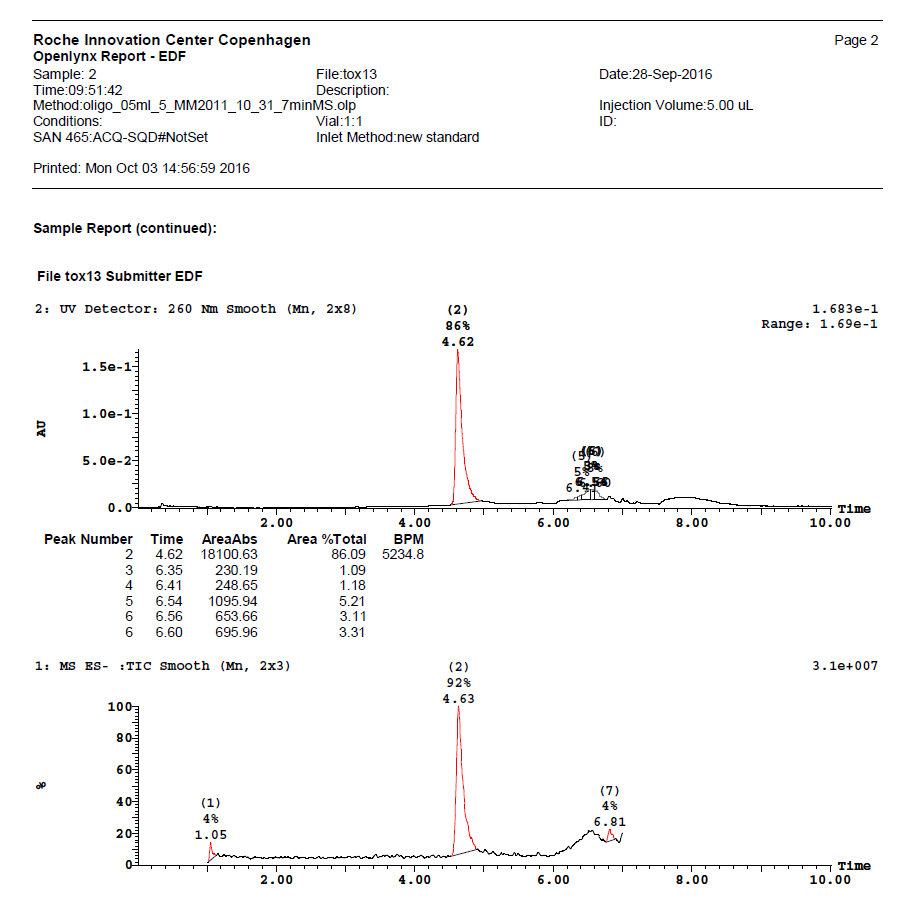


A14 (Tox14)


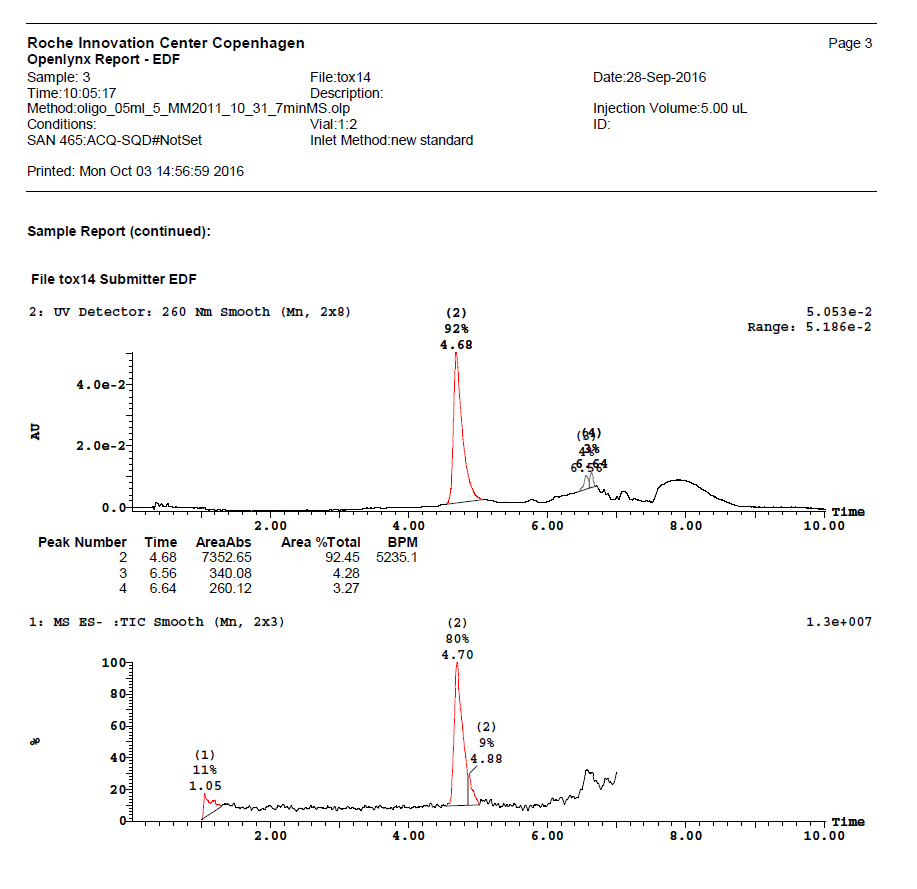


A15 (Tox15)


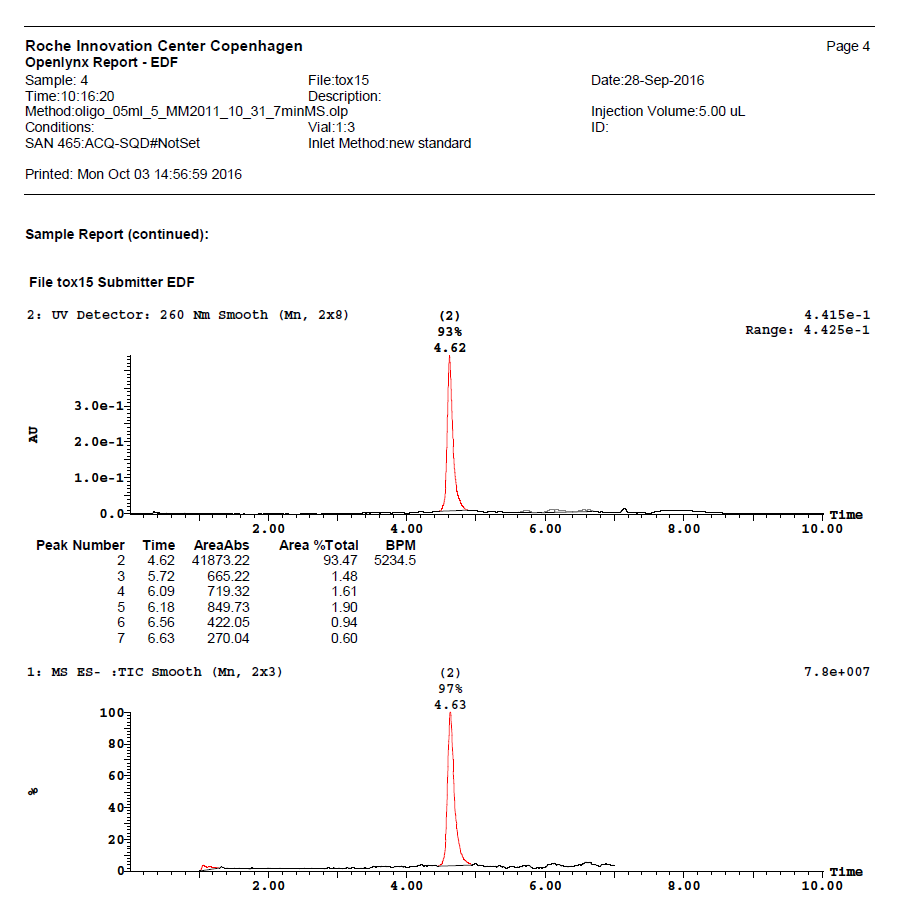


A16 (Tox16)


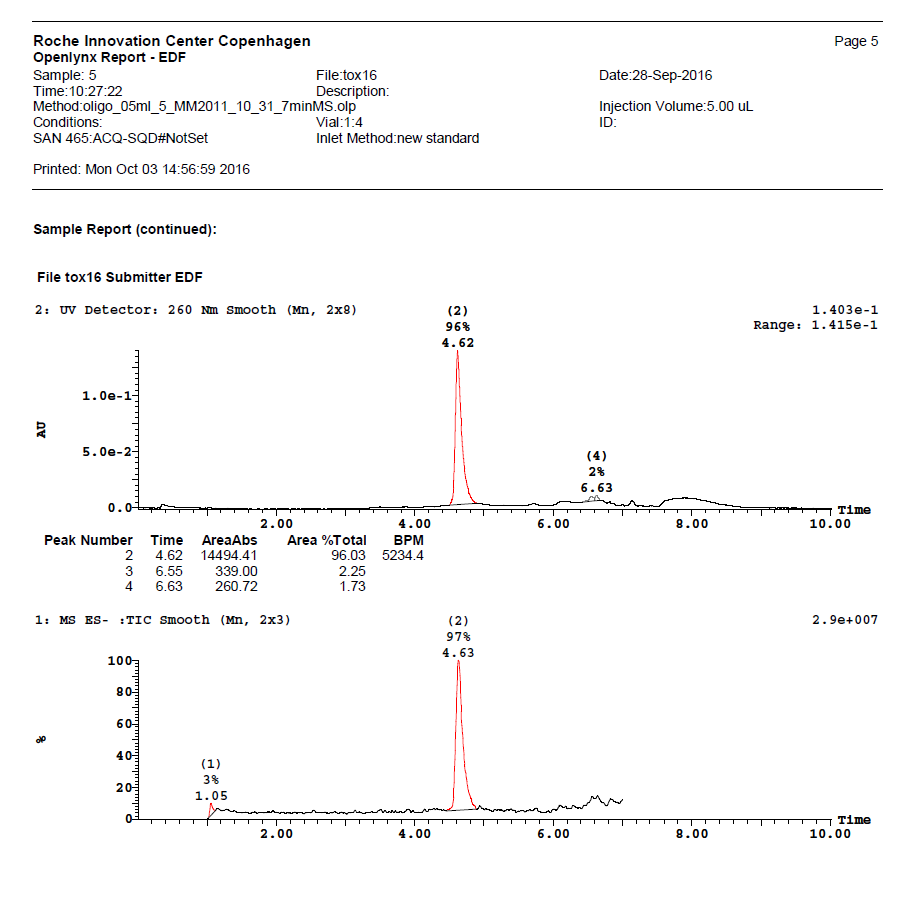


B1 (Tox1b)


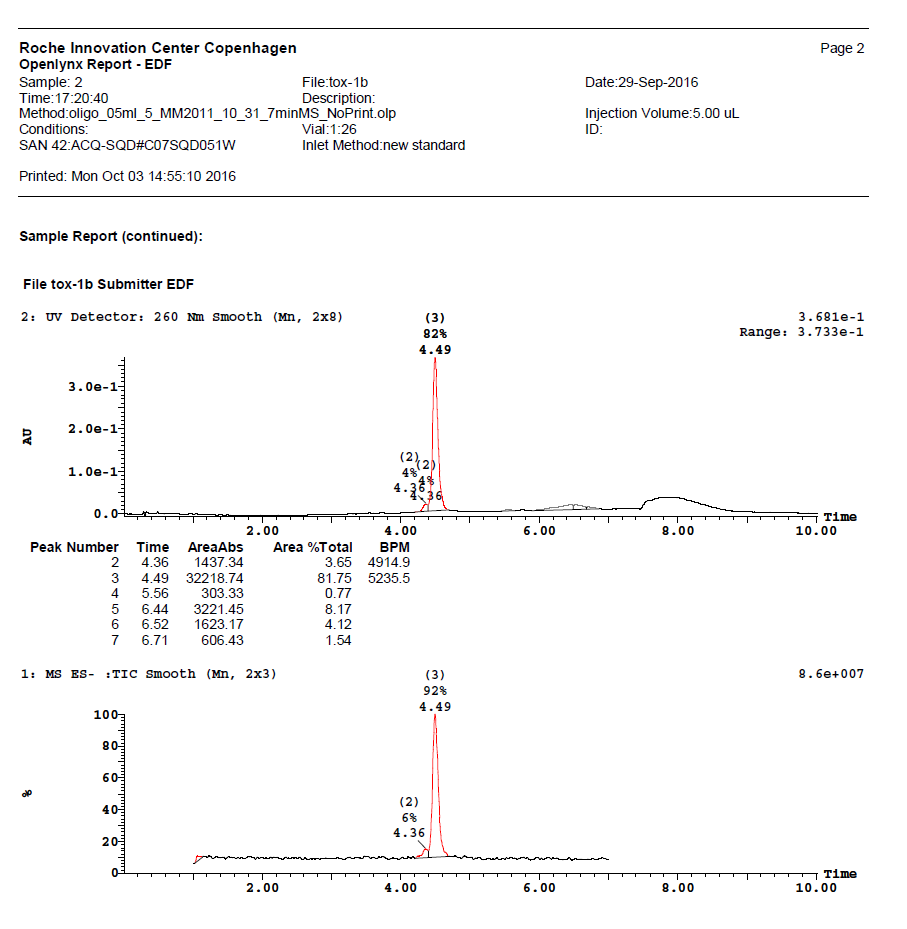


B2 (Tox2b)


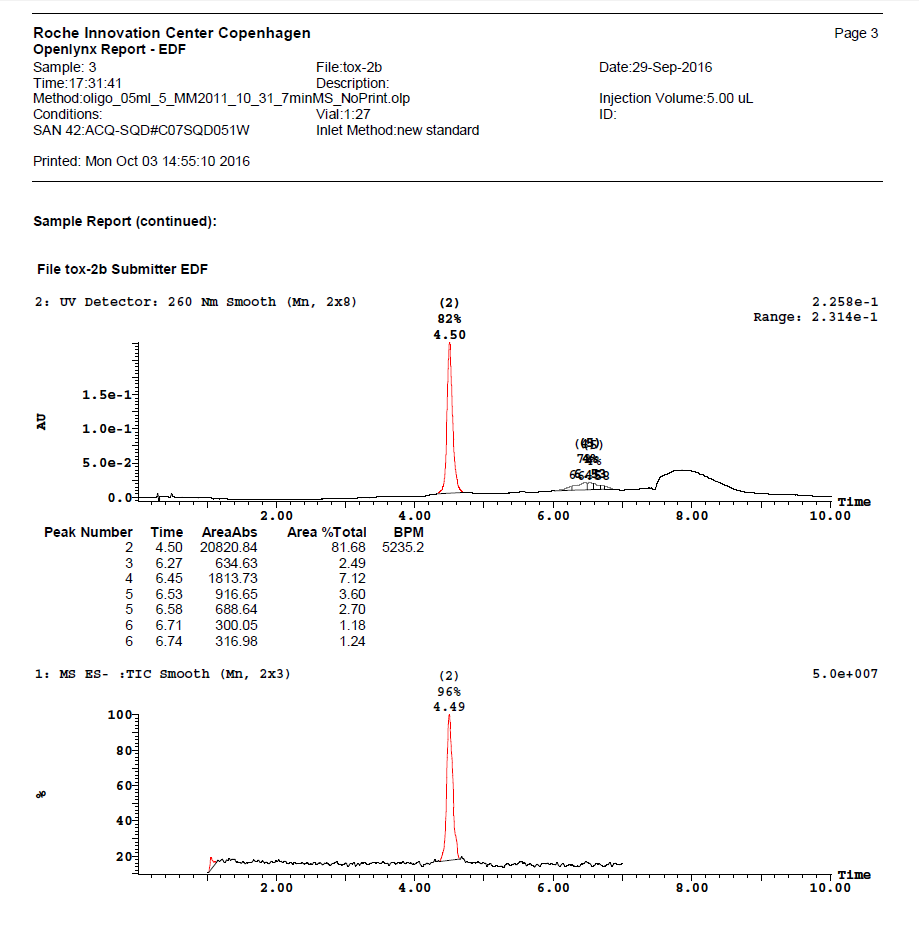


B3 (Tox3b)


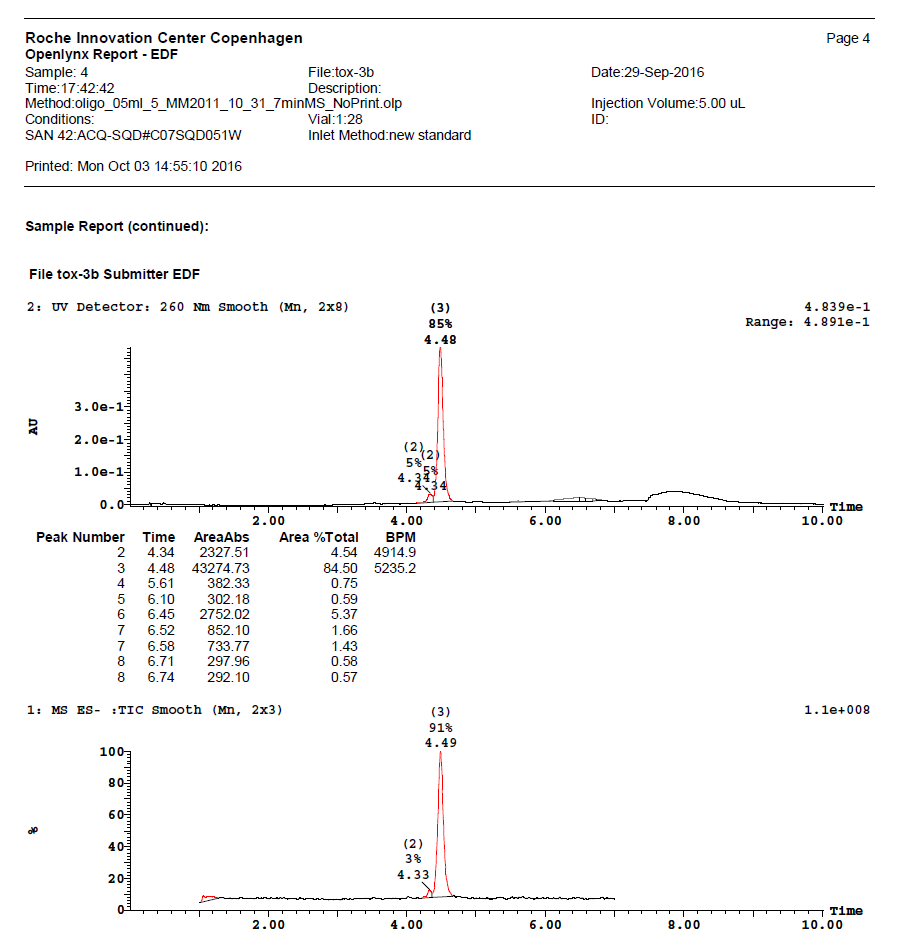


B4 (Tox4b)


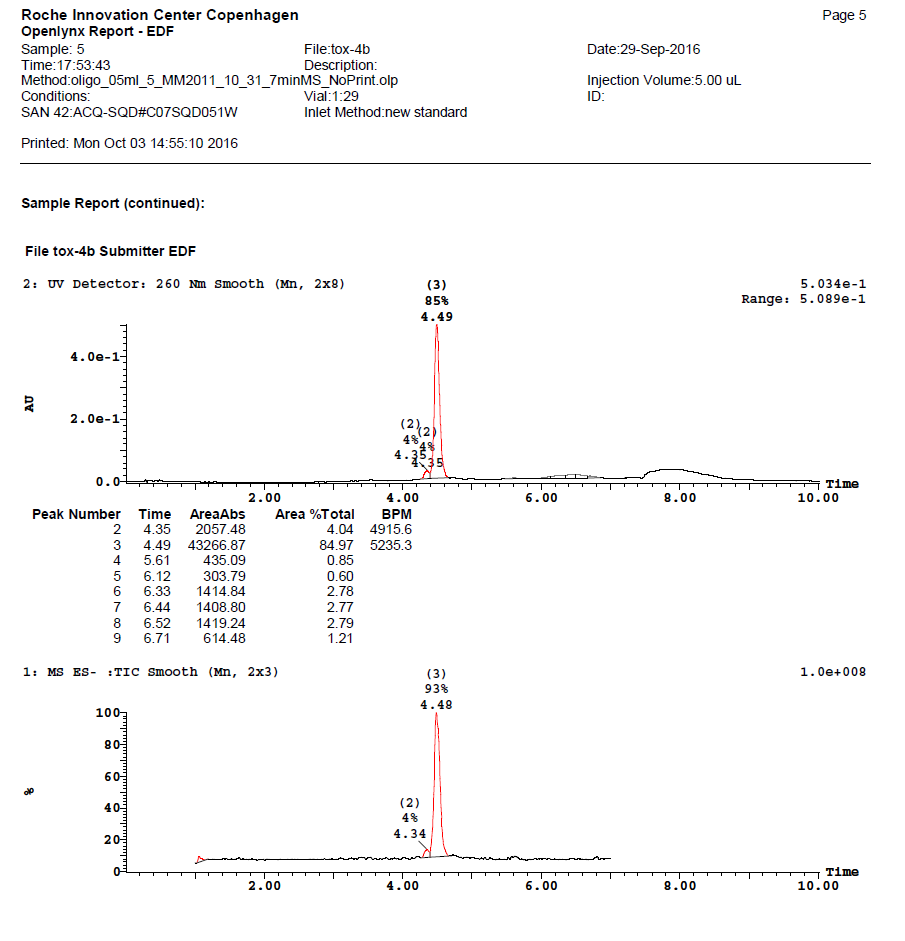


B5 (Tox5b)


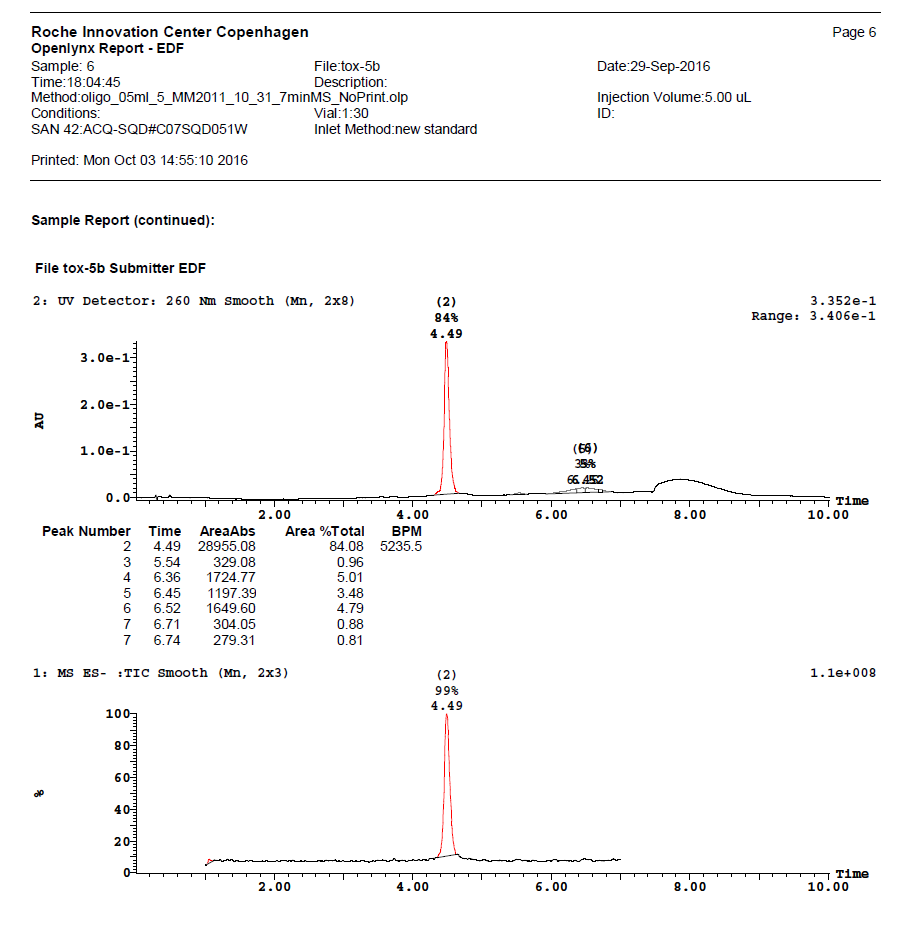


B6 (Tox6b)


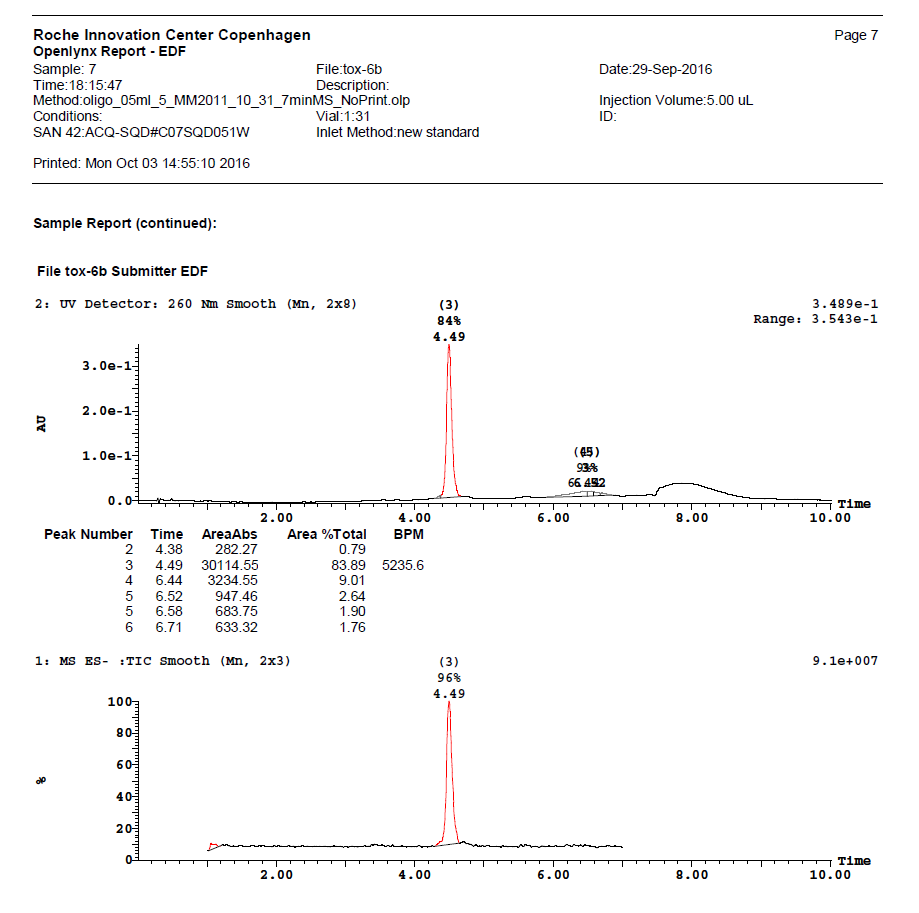


B7 (Tox7b)


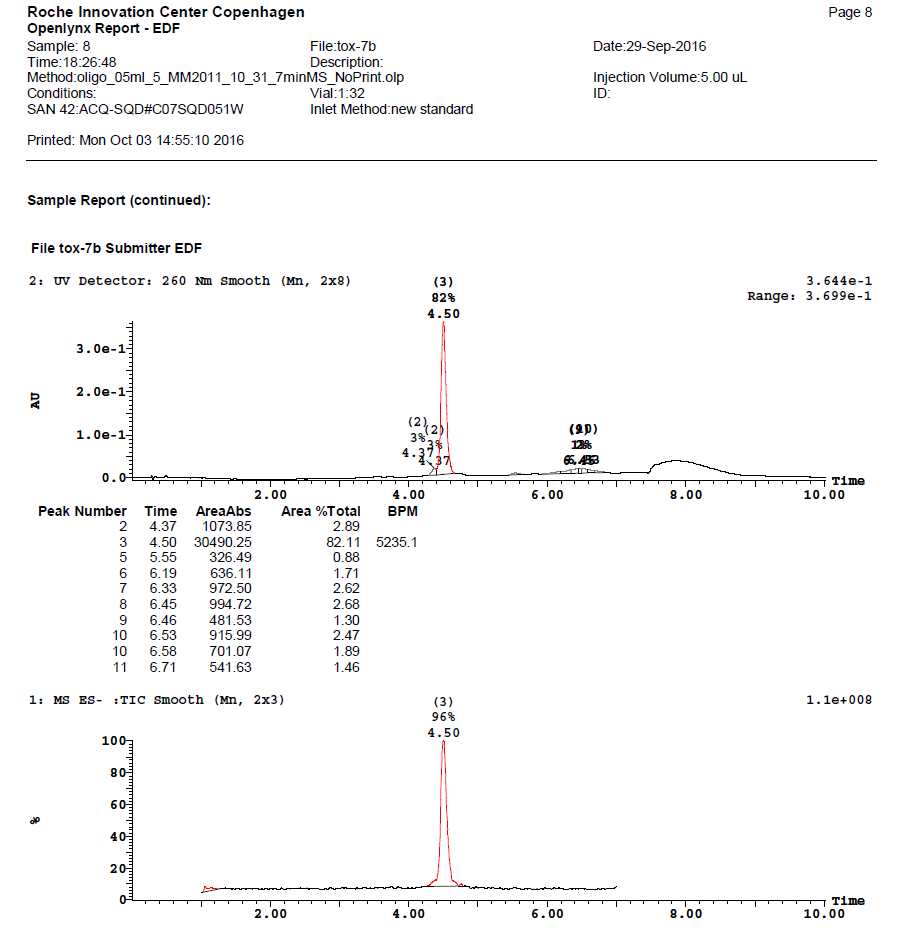


B8 (Tox8b)


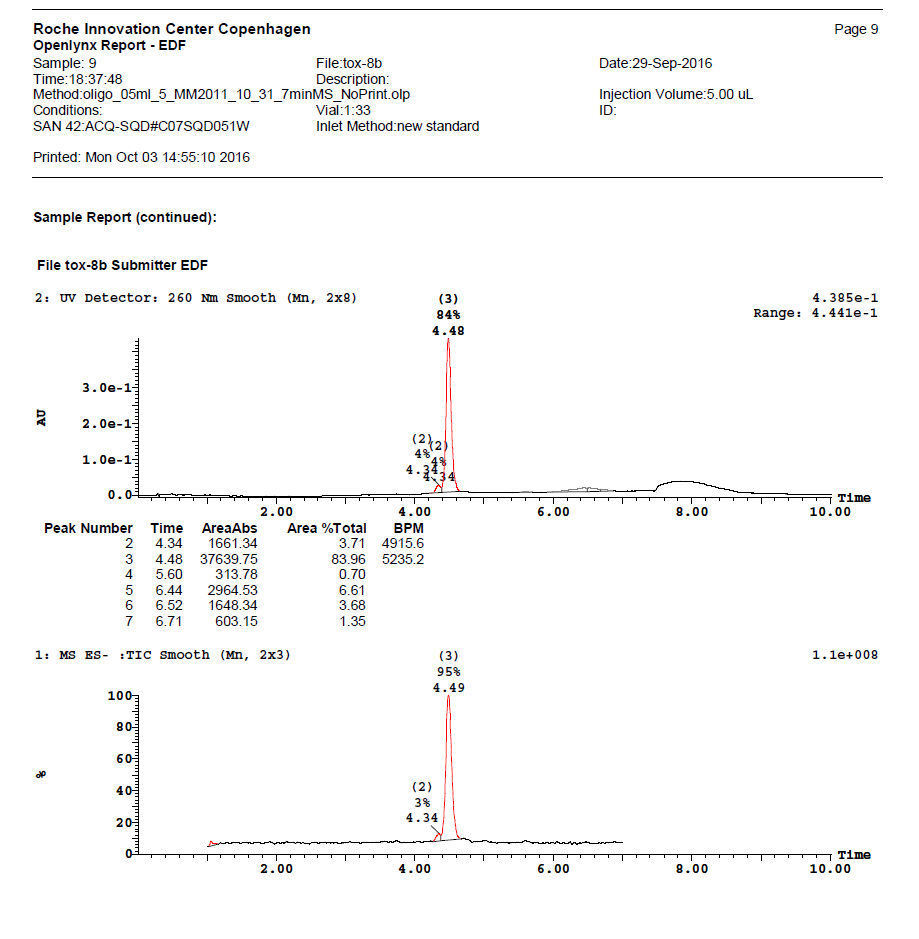


B9 (Tox9b)


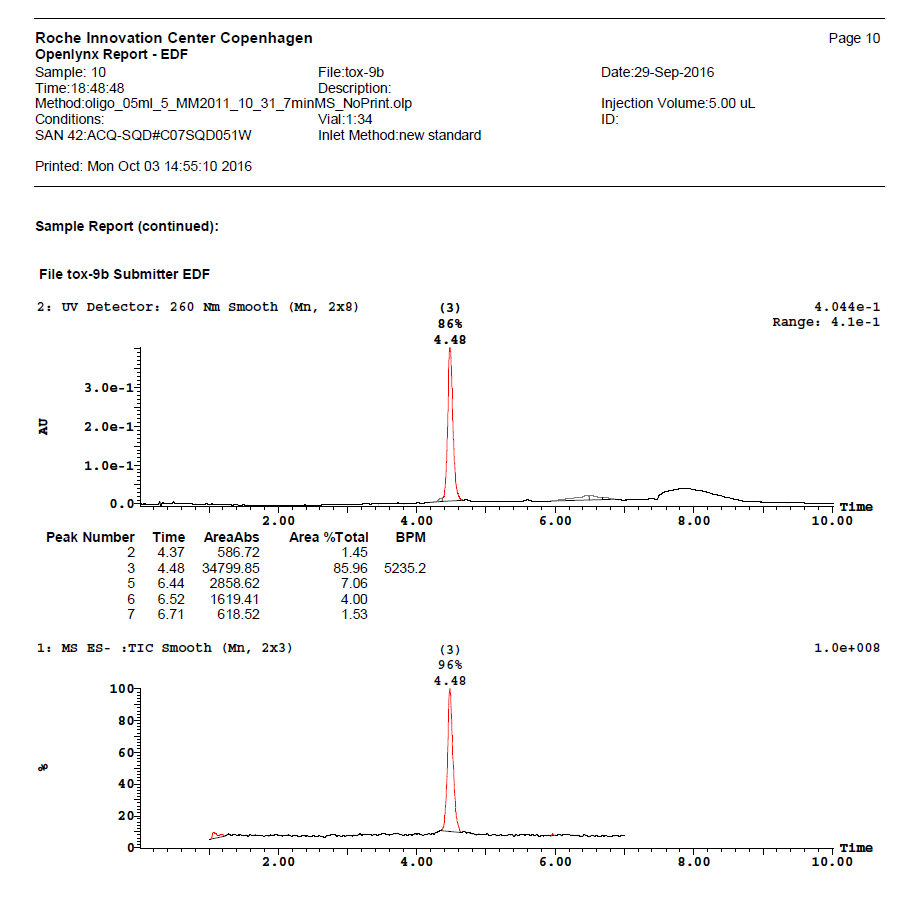


B10 (10b)


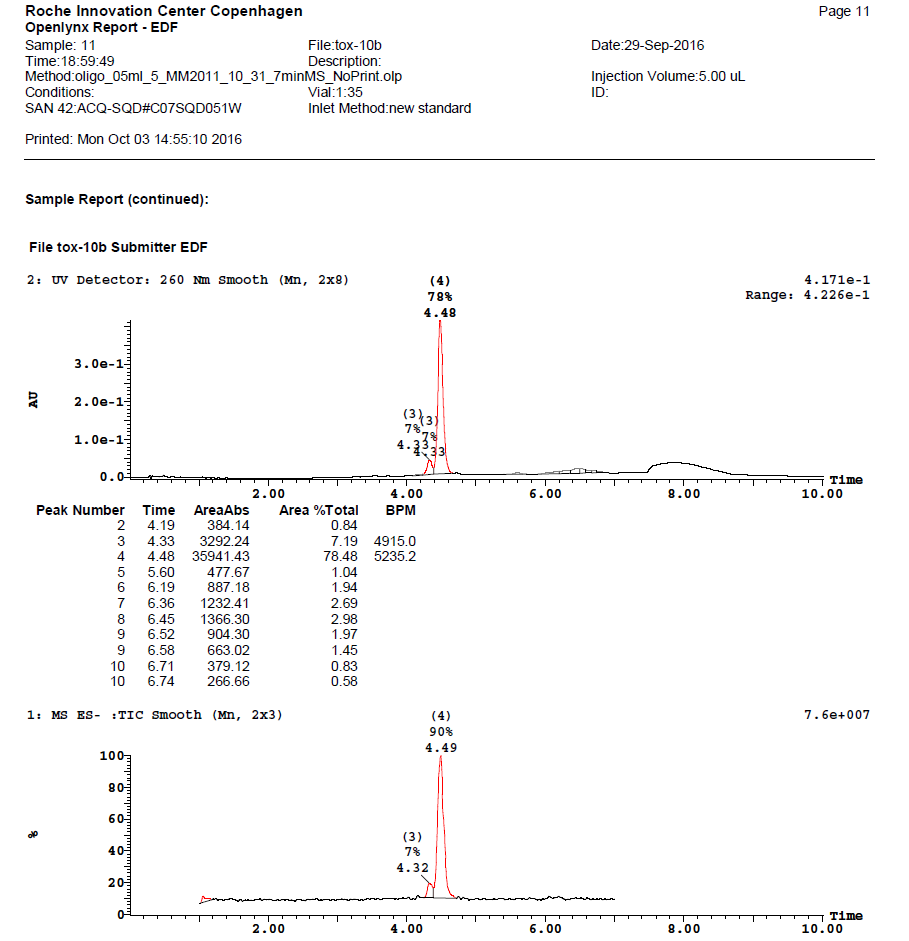


B11 (Tox11b)


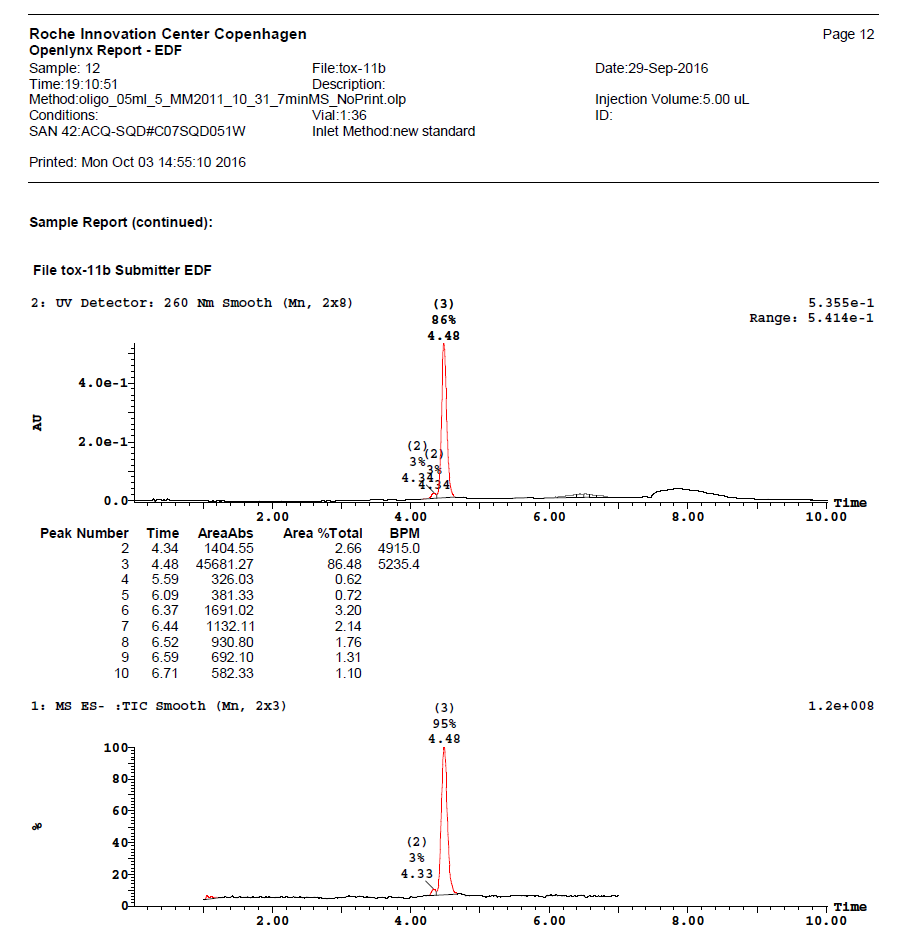


B12 (Tox12b)


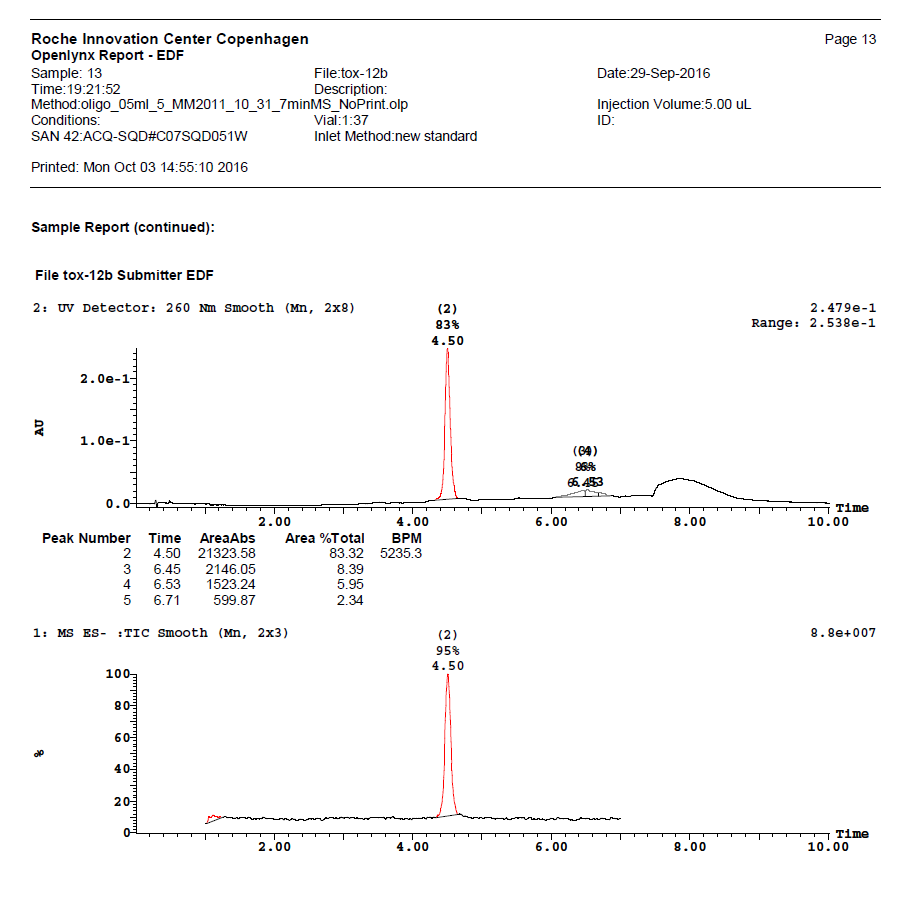


B13 (Tox13b)


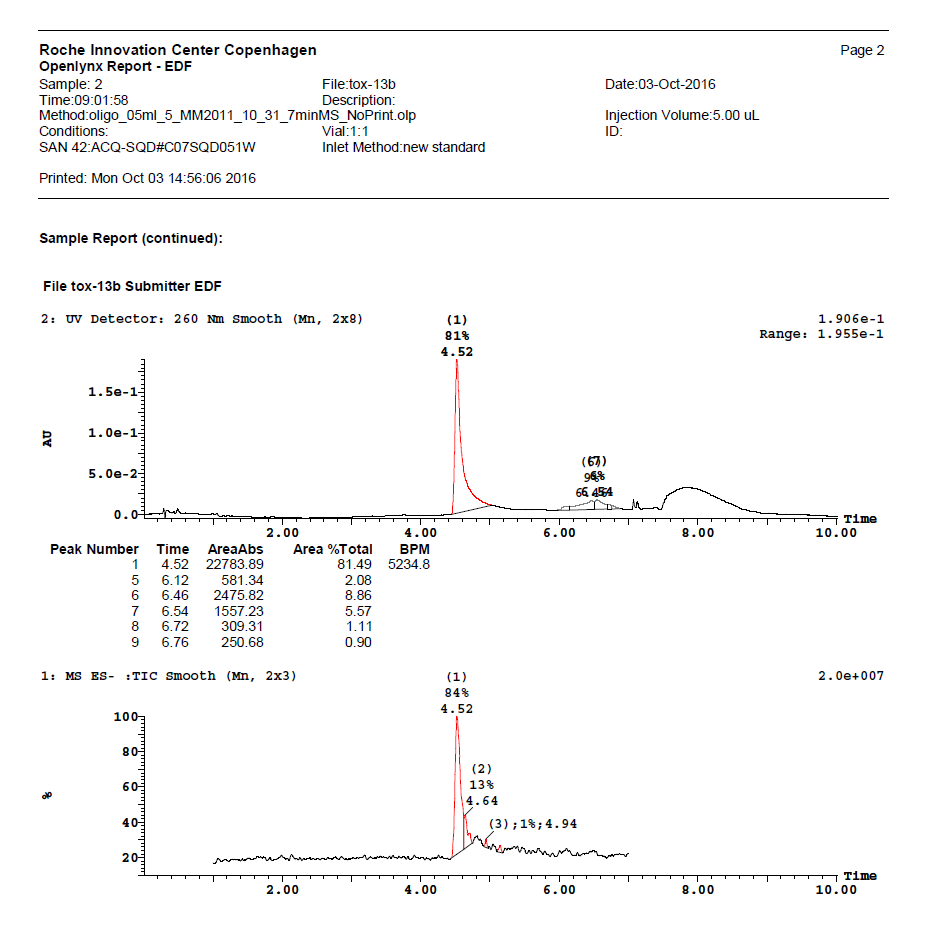


B14 (Tox14b)


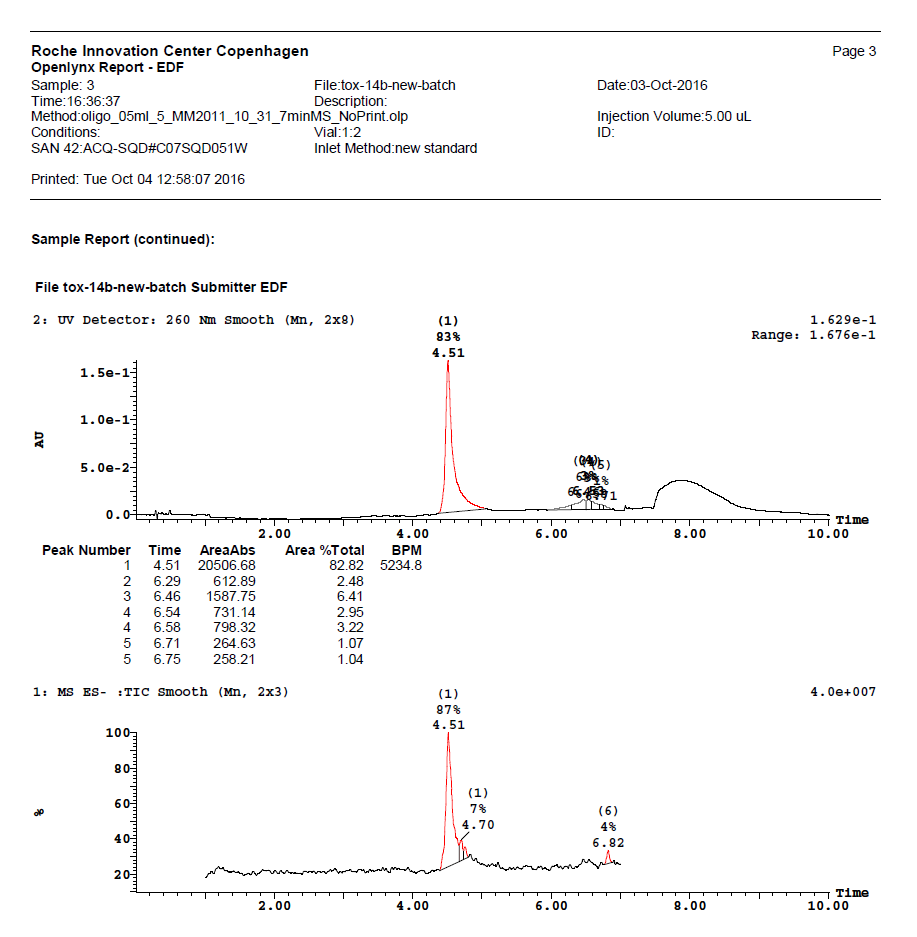


B15 (Tox15b)


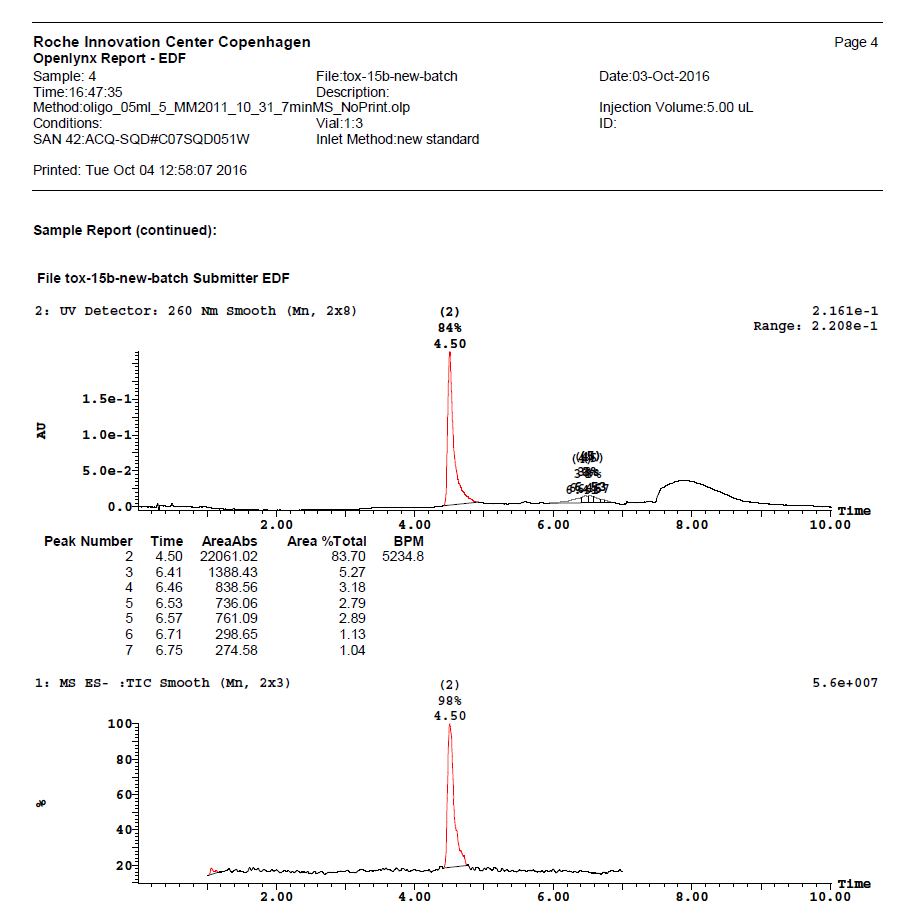


B16 (Tox16b)


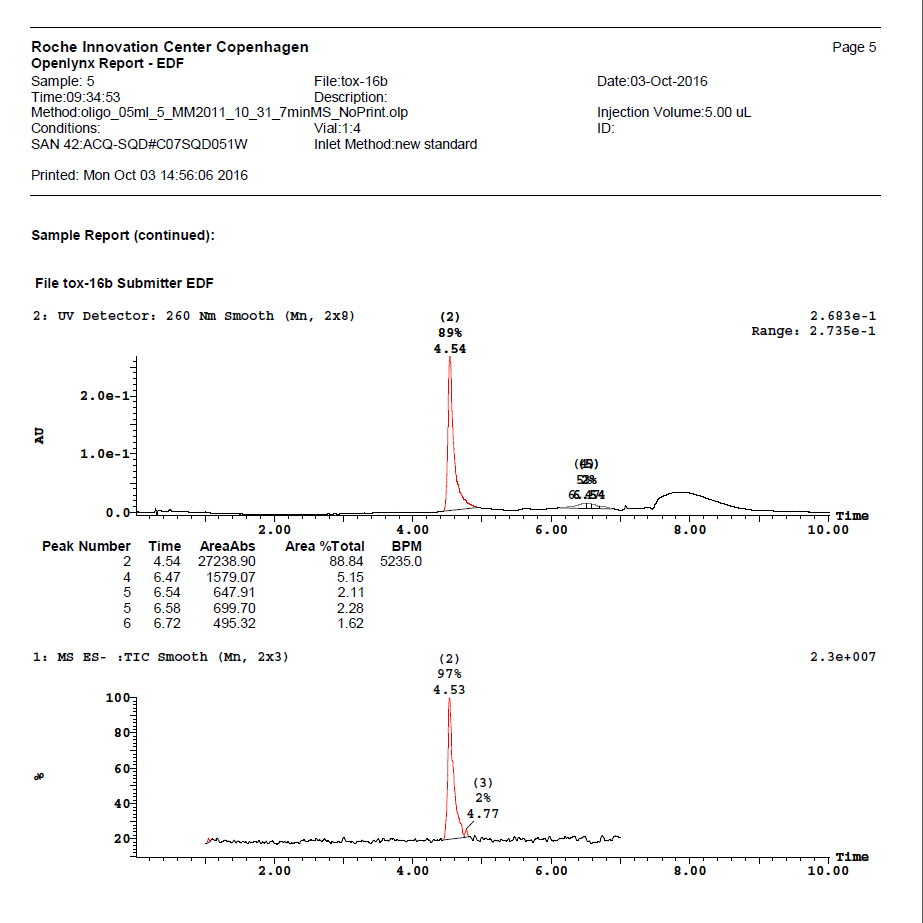


|  |  | **Oligo** | **Purity above*** | **Calculated molecular mass** | **Found molecular mass** |
| --- | --- | --- | --- | --- | --- |
| **1** | [**A**](http://apex.pez.roche.com/ricc/f?p=118:25:3303459166149::NO:RP,25:P25_BATCH_ID:94175) | **Tox1** | **83** | **5234.1 g/mol** | **5235.5 g/mol** |
| **2** | **A2** | **Tox2** | **78** | **5234.1 g/mol** | **5235.3 g/mol** |
| **3** | **A3** | **Tox3** | **89** | **5234.1 g/mol** | **5235.1 g/mol** |
| **4** | **A4** | **Tox4** | **84** | **5234.1 g/mol** | **5235.5 g/mol** |
| **5** | **A5** | **Tox5** | **76** | **5234.1 g/mol** | **5235.6 g/mol** |
| **6** | **A6** | **Tox6** | **81** | **5234.1 g/mol** | **5235.4 g/mol** |
| **7** | **A7** | **Tox7** | **61** | **5234.1 g/mol** | **5235.2 g/mol** |
| **8** | **A8** | **Tox8** | **82** | **5234.1 g/mol** | **5235.4 g/mol** |
| **9** | **A9** | **Tox9** | **83** | **5234.1 g/mol** | **5236.1 g/mol** |
| **10** | **A10** | **Tox10** | **88** | **5234.1 g/mol** | **5235.3 g/mol** |
| **11** | **A11** | **Tox11** | **85** | **5234.1 g/mol** | **5235.3 g/mol** |
| **12** | **A12** | **Tox12** | **78** | **5234.1 g/mol** | **5235.7 g/mol** |
| **13** | **A13** | **Tox13** | **86** | **5234.1 g/mol** | **5234.8 g/mol** |
| **14** | **A14** | **Tox14** | **92** | **5234.1 g/mol** | **5235.1 g/mol** |
| **15** | **A15** | **Tox15** | **93** | **5234.1 g/mol** | **5234.5 g/mol** |
| **16** | **A16** | **Tox16** | **96** | **5234.1 g/mol** | **5234.4 g/mol** |
| **17** | **B1** | **Tox1b** | **82** | **5234.1 g/mol** | **5235.5 g/mol** |
| **18** | **B2** | **Tox2b** | **82** | **5234.1 g/mol** | **5235.2 g/mol** |
| **19** | **B3** | **Tox3b** | **85** | **5234.1 g/mol** | **5235.2 g/mol** |
| **20** | **B4** | **Tox4b** | **85** | **5234.1 g/mol** | **5235.3 g/mol** |
| **21** | **B5** | **Tox5b** | **84** | **5234.1 g/mol** | **5235.5 g/mol** |
| **22** | **B6** | **Tox6b** | **84** | **5234.1 g/mol** | **5235.6 g/mol** |
| **23** | **B7** | **Tox7b** | **82** | **5234.1 g/mol** | **5235.1 g/mol** |
| **24** | **B8** | **Tox8b** | **84** | **5234.1 g/mol** | **5235.2 g/mol** |
| **25** | **B9** | **Tox9b** | **86** | **5234.1 g/mol** | **5235.2 g/mol** |
| **26** | **B10** | **Tox10b** | **78** | **5234.1 g/mol** | **5235.2 g/mol** |
| **27** | **B11** | **Tox11b** | **86** | **5234.1 g/mol** | **5235.4 g/mol** |
| **28** | **B12** | **Tox12b** | **83** | **5234.1 g/mol** | **5235.3 g/mol** |
| **29** | **B13** | **Tox13b** | **81** | **5234.1 g/mol** | **5234.8 g/mol** |
| **30** | **B14** | **Tox14b** | **83** | **5234.1 g/mol** | **5234.8 g/mol** |
| **31** | **B15** | **Tox15b** | **84** | **5234.1 g/mol** | **5234.8 g/mol** |
| **32** | **B16** | **Tox16b** | **89** | **5234.1 g/mol** | **5235.0 g/mol** |

- **The stated purity is the minimum purity as the peak after 6 min (also present in the blank run) in some cases also is taken into account using the automated integration.**

**Resynthesis of 11 selected compounds from pattern 1 and pattern 2: UV (260 nm) and MS ES- is provided + blank run (Milli-Q® water injected)**

**Representative sample of background: “blank run” using 2 µL Milli-Q® water as the injected sample.**

**The sample chromatograms have been cropped in order to hide internal reference numbers for the compounds.**

**
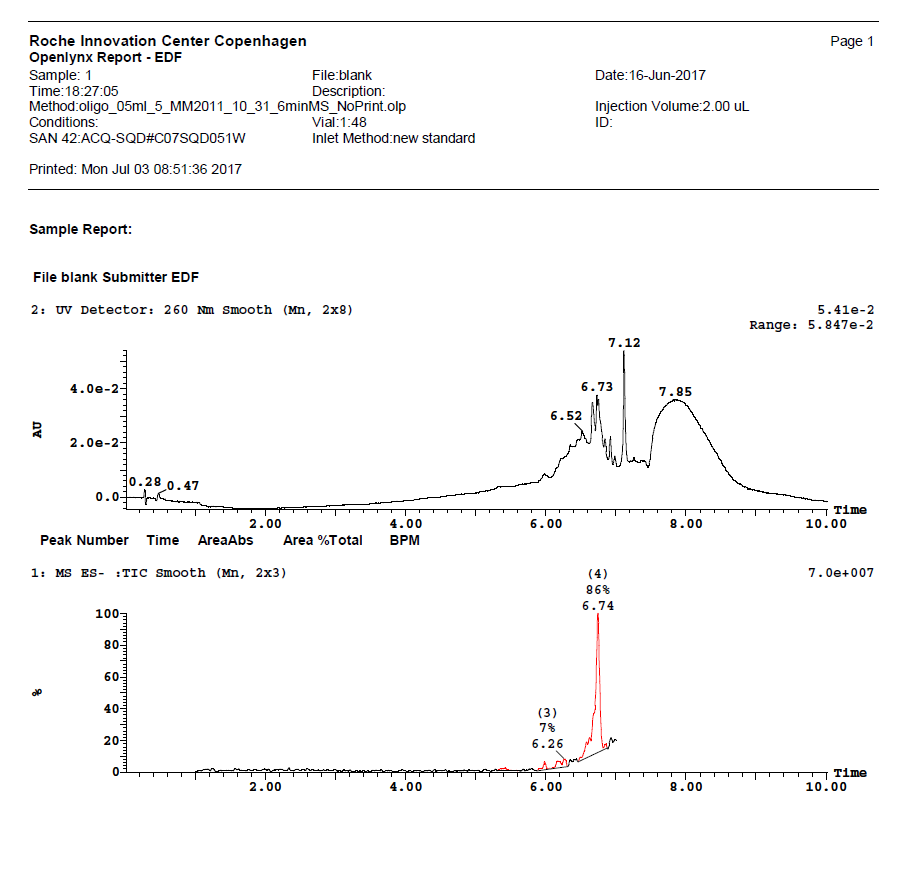
**

**B9 (tox9)**

**
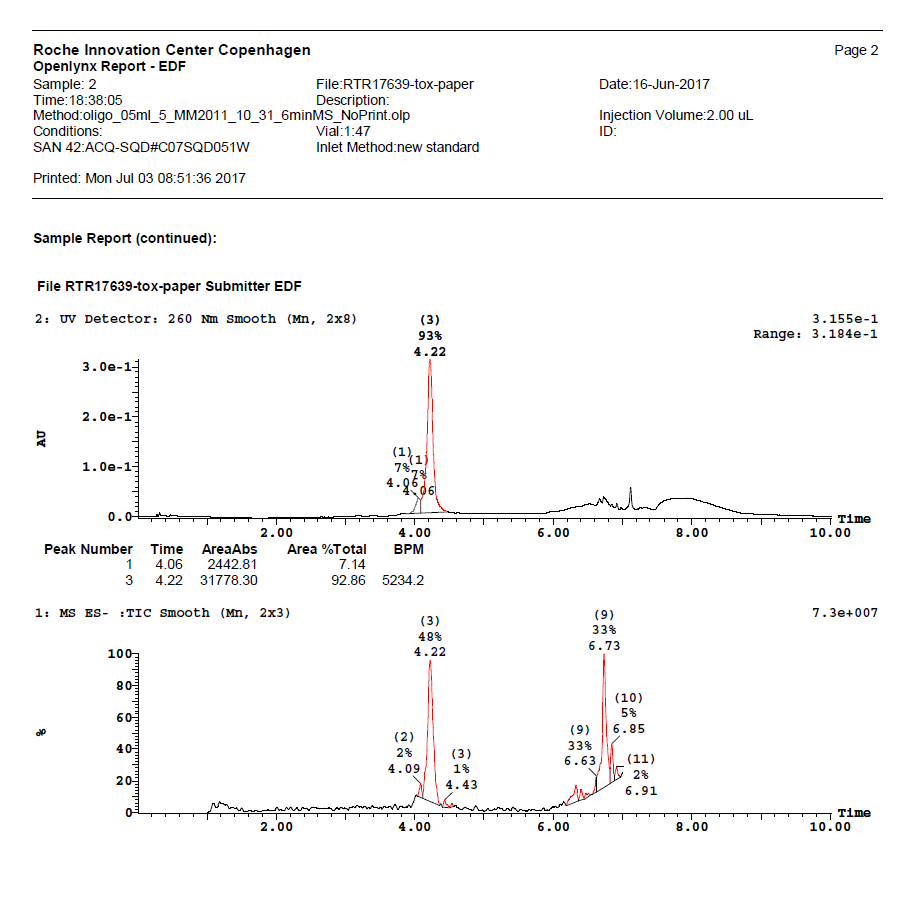
**

**B15 (Tox15b)**

**
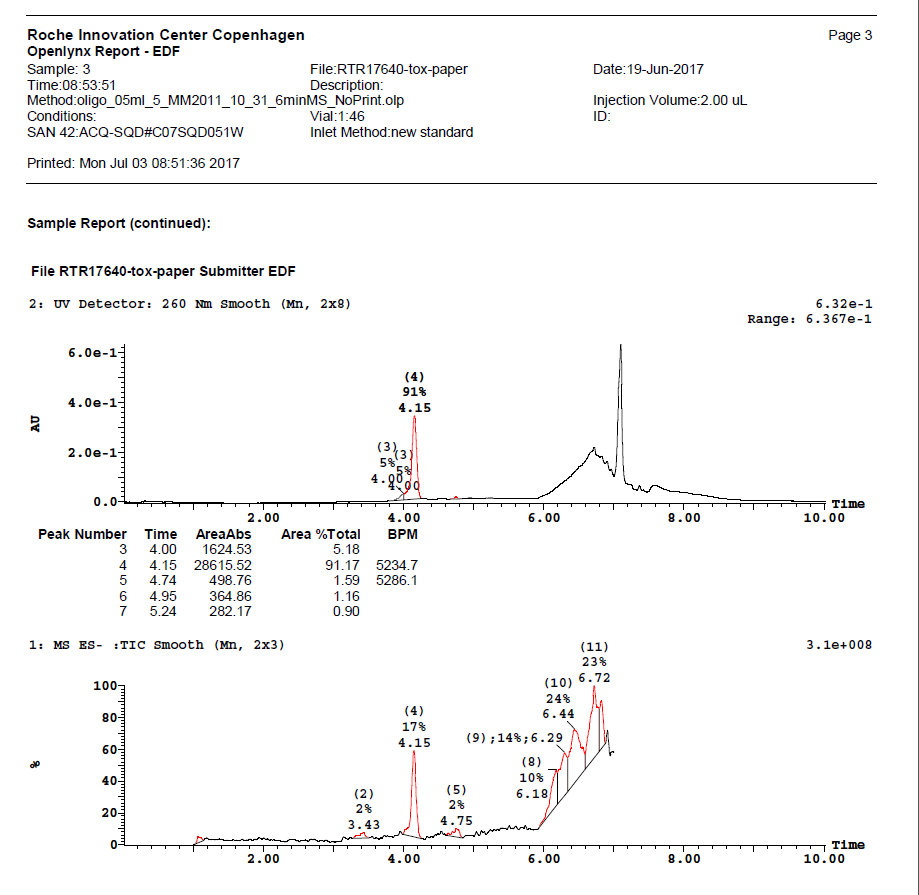
**

**A6 (Tox6)**

**
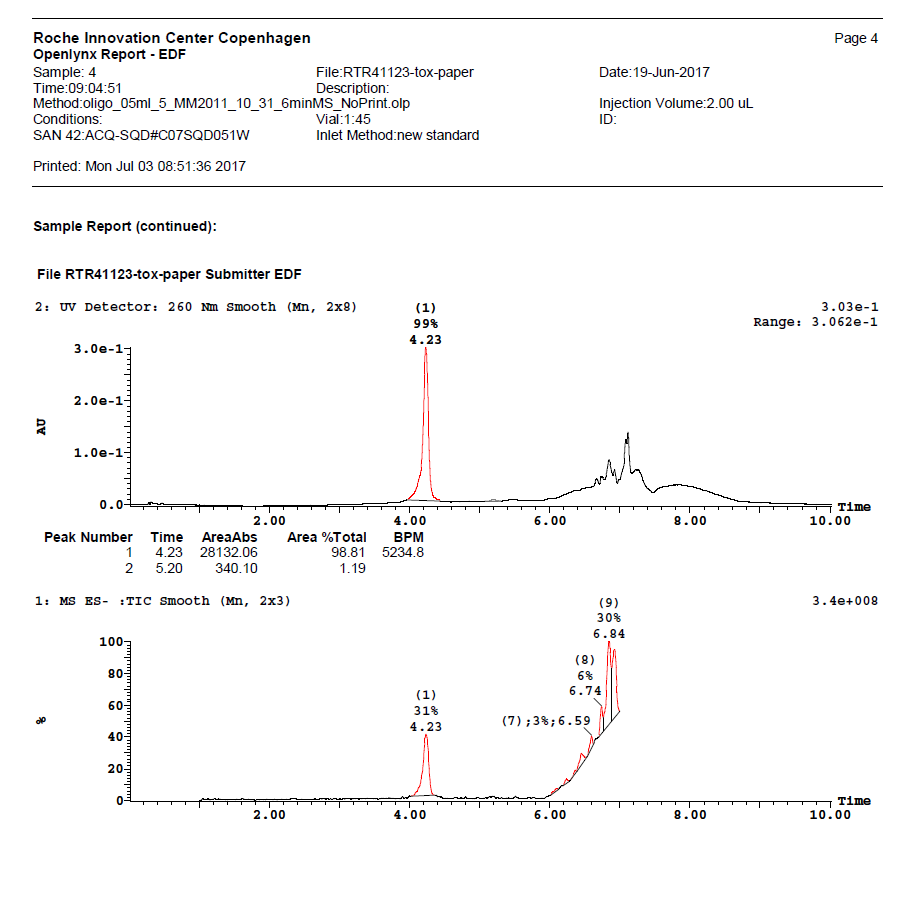
**

**A8 (Tox8)**

**
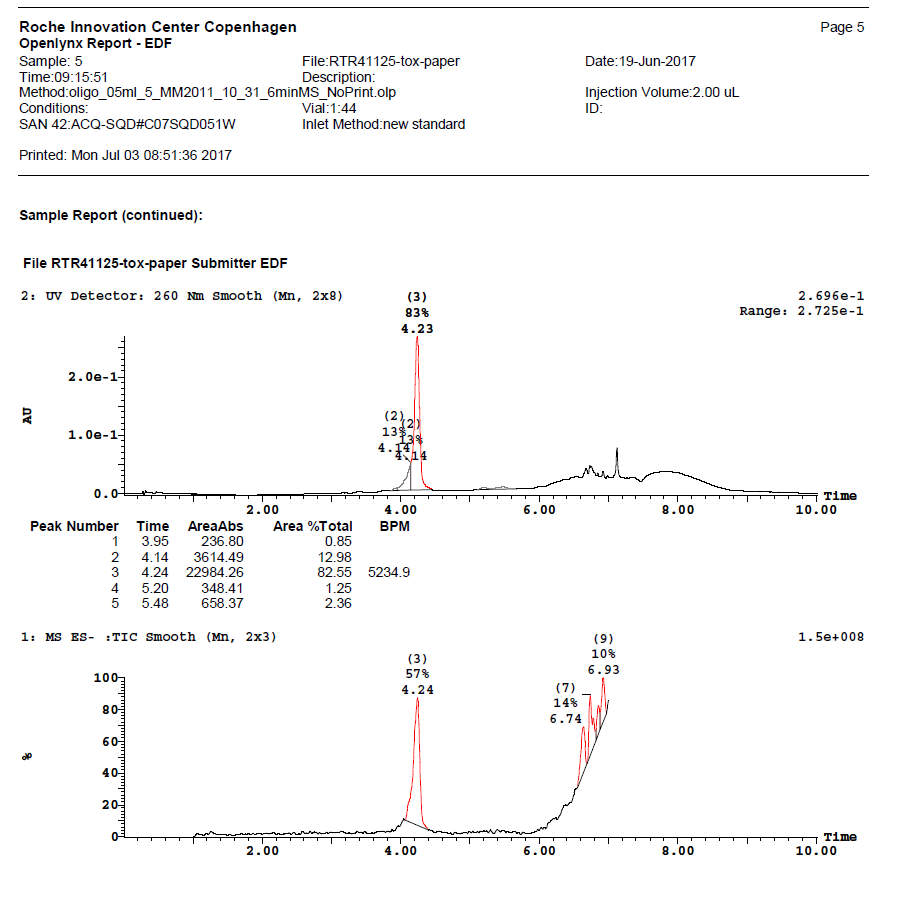
**

**A11 (Tox11)**

**
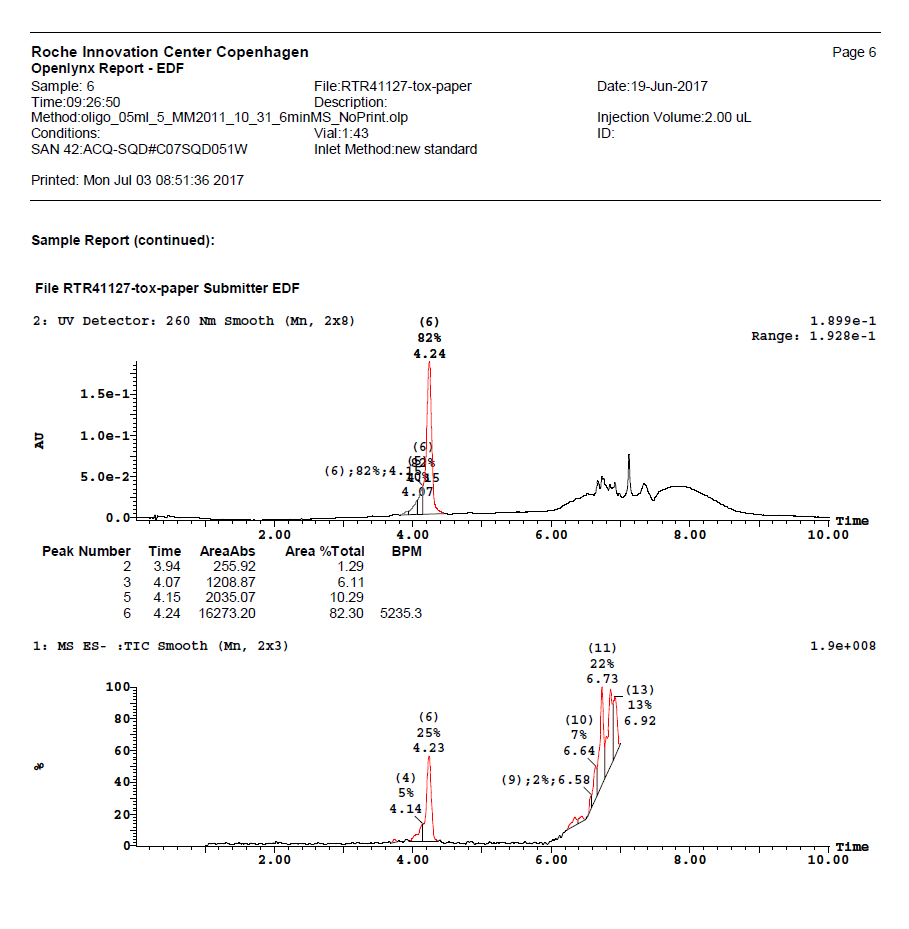
**

**A14 (Tox14)**

**
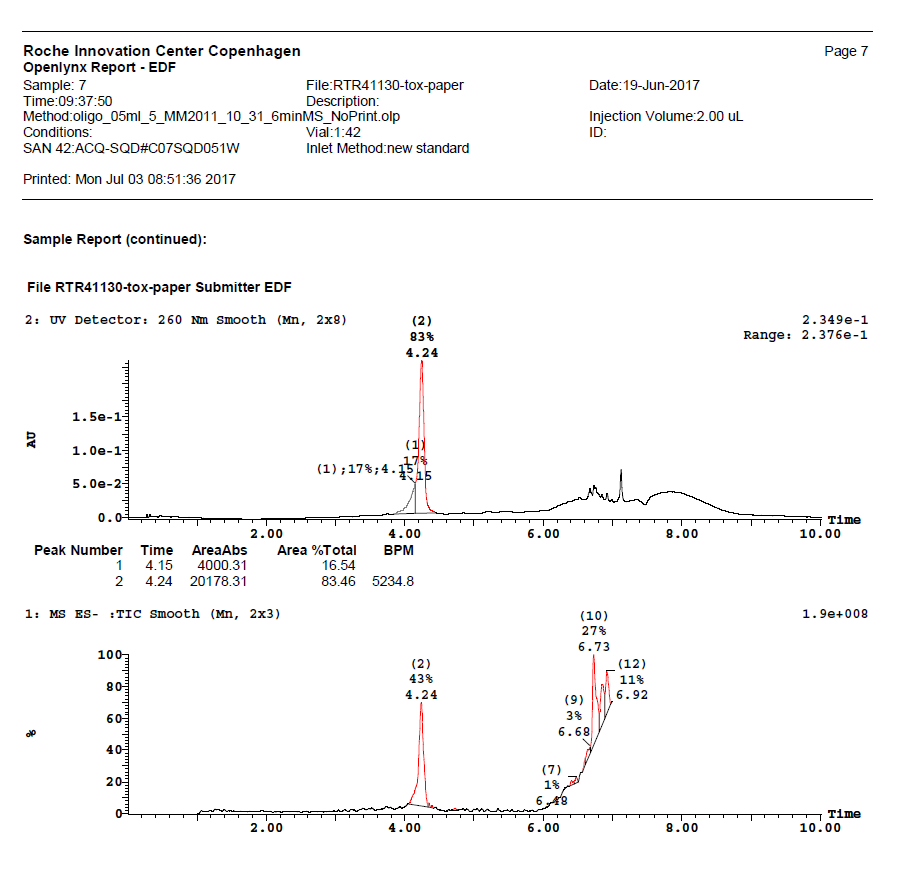
**

**B4 (Tox4b)**

**
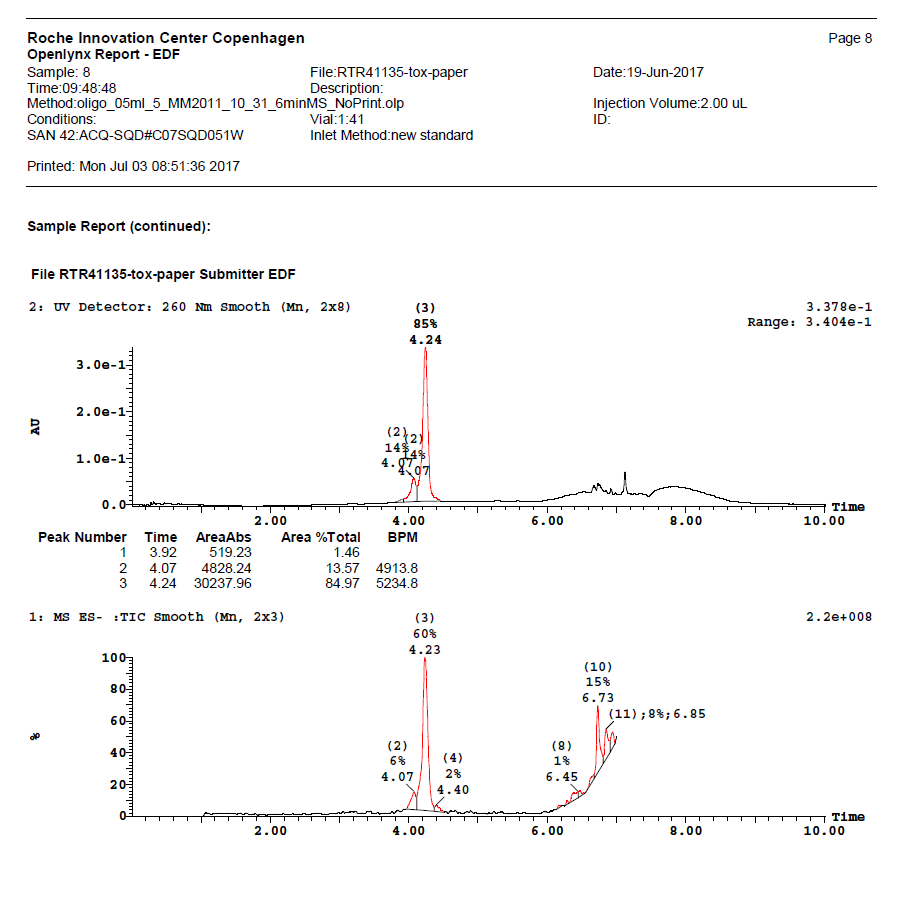
**

**B10 (Tox10b)**

**
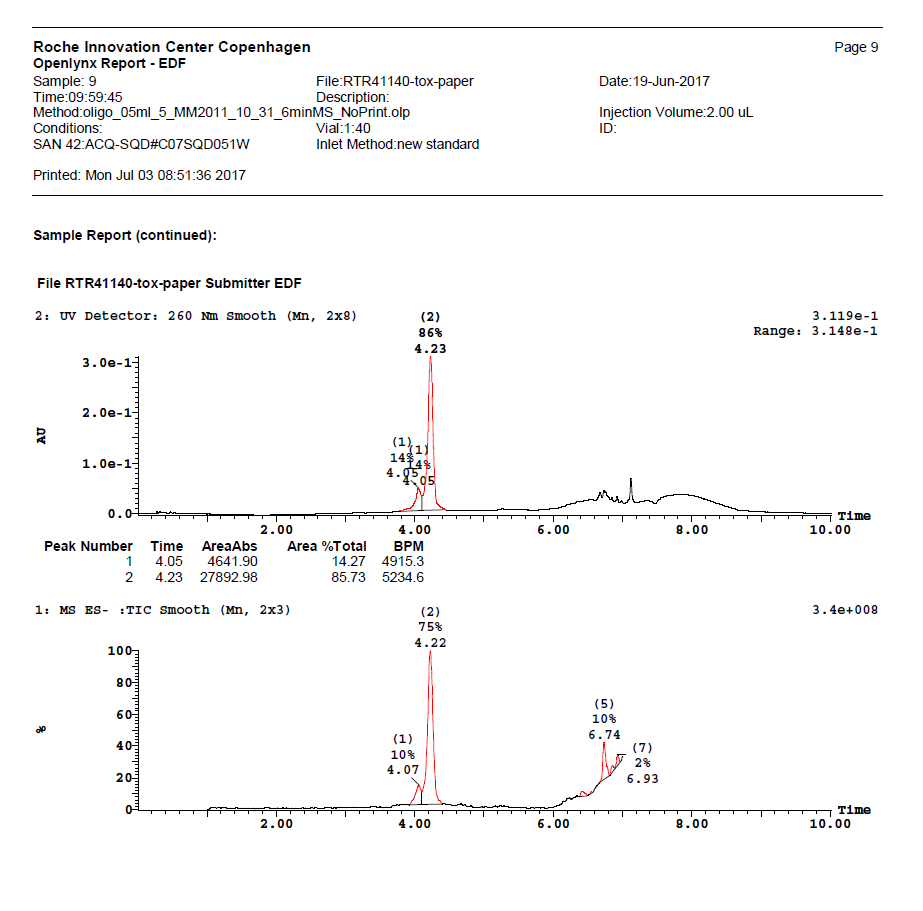
**

**B13 (Tox13b)**

**
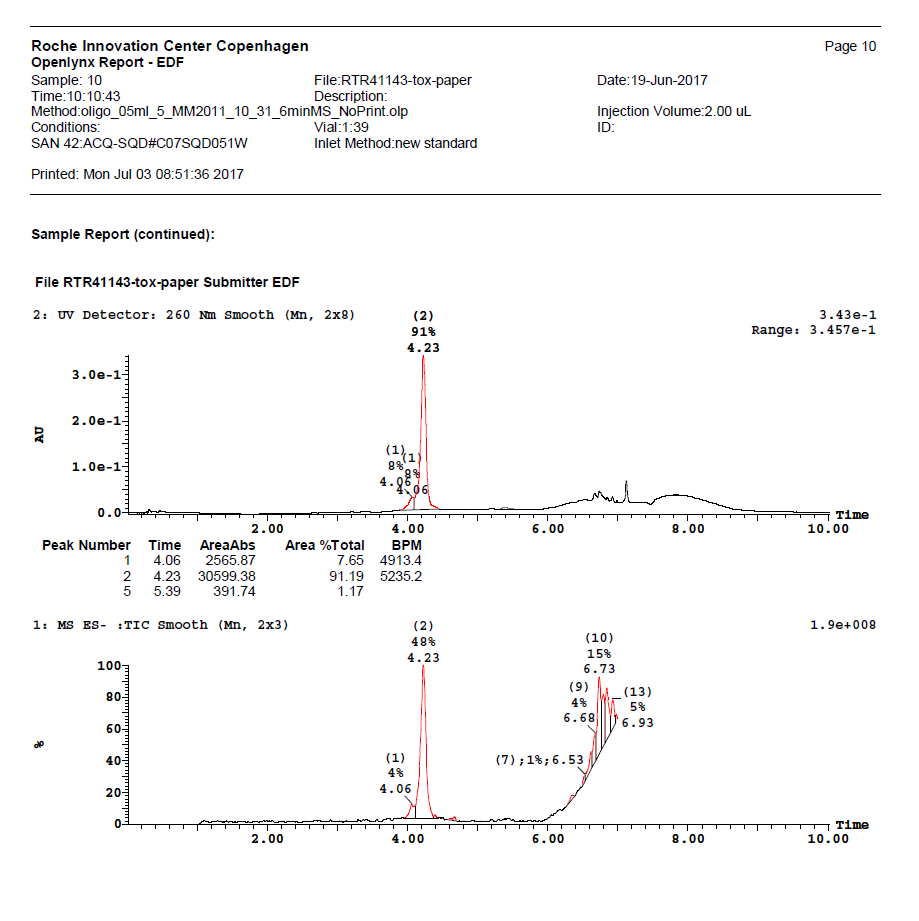
**

**B14 (Tox14b)**

**
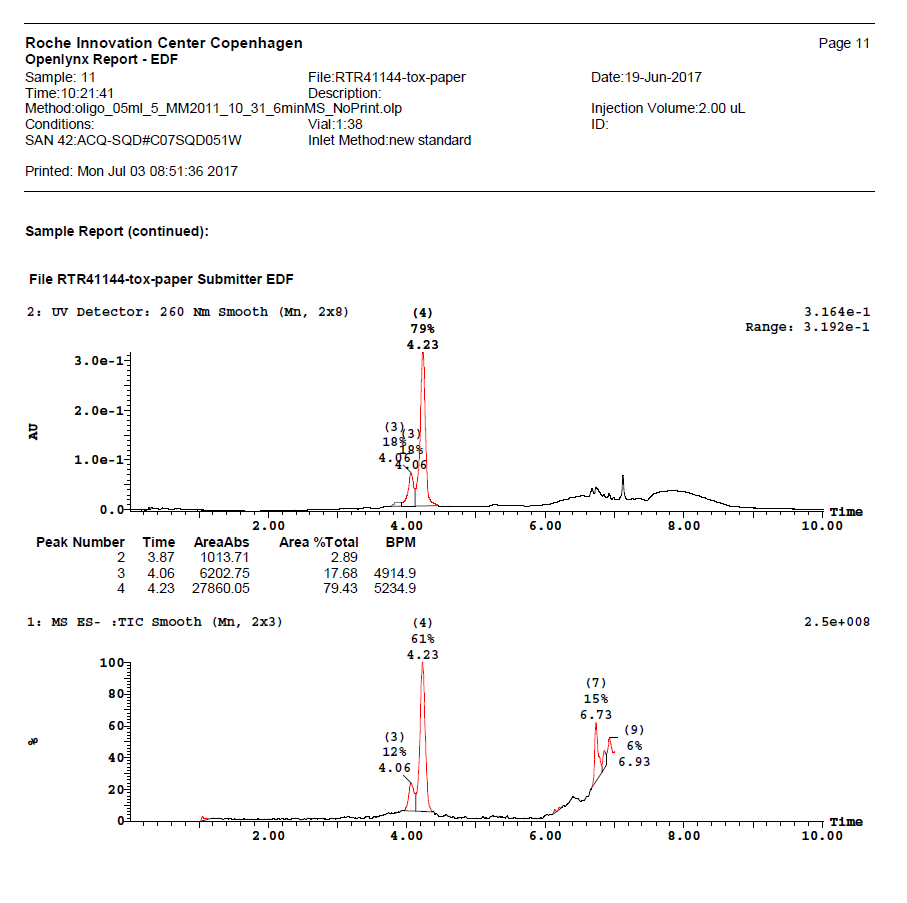
**

**B16 (Tox16b)**


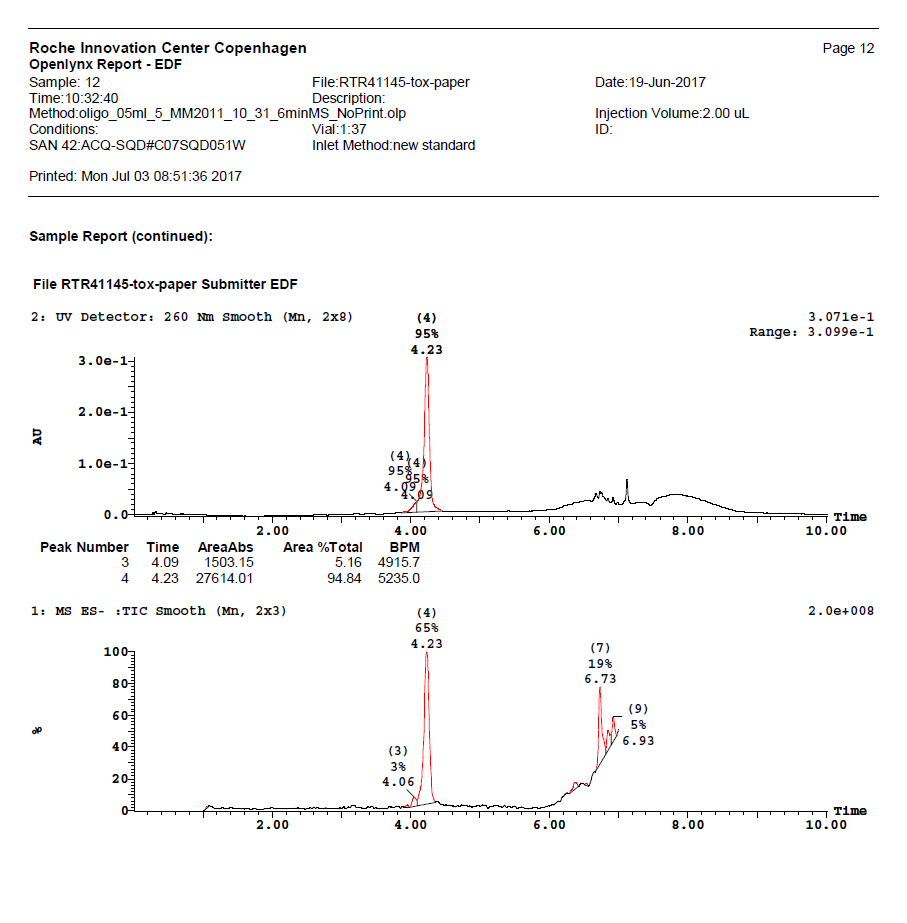


|  |  | **Oligo** | **Purity** | **Calculated molecular mass** | **Found molecular mass** |
| --- | --- | --- | --- | --- | --- |
| **1** | **B9** | **Tox9b** | **93** | **5234.1 g/mol** | **5234.2 g/mol** |
| **2** | **B15** | **Tox15b** | **91** | **5234.1 g/mol** | **5234.7 g/mol** |
| **3** | **A6** | **Tox6** | **99** | **5234.1 g/mol** | **5234.8 g/mol** |
| **4** | **A8** | **Tox8** | **83** | **5234.1 g/mol** | **5234.9 g/mol** |
| **5** | **A11** | **Tox11** | **82** | **5234.1 g/mol** | **5235.3 g/mol** |
| **6** | **A14** | **Tox14** | **83** | **5234.1 g/mol** | **5234.8 g/mol** |
| **7** | **B4** | **Tox4b** | **85** | **5234.1 g/mol** | **5234.8 g/mol** |
| **8** | **B10** | **Tox10b** | **86** | **5234.1 g/mol** | **5234.6 g/mol** |
| **9** | **B13** | **Tox13b** | **91** | **5234.1 g/mol** | **5235.2 g/mol** |
| **10** | **B14** | **Tox14b** | **79** | **5234.1 g/mol** | **5234.9 g/mol** |
| **11** | **B16** | **Tox16b** | **95** | **5234.1 g/mol** | **5235.0 g/mol** |

**In vitro safety of initial 32 compounds**

S1 - In vitro toxicity of stereodefined LNA sublibraries expressed as % assay window (% AW) at 30 µM in mouse hepatocytes (hepatotox)and 100 µM in human PTEC TERT1 cells (renal tox).

| **Compound** | **Hepatotox**  **LDH % AW** | **Hepatotox**  **ATP % AW** | **Renal Tox**  **EGF % AW** |
| --- | --- | --- | --- |
| **C1** | 110 | 95 | 56 |
| **A1** | 69 | 65 | 19 |
| **A2** | 56 | 72 | 30 |
| **A3** | 63 | 82 | 7 |
| **A4** | 40 | 70 | 18 |
| **A5** | 81 | 79 | 4 |
| **A6** | 126 | 103 | 5 |
| **A7** | 54 | 67 | 4 |
| **A8** | 34 | 57 | 6 |
| **A10** | 42 | 71 | 3 |
| **A11** | 77 | 144 | 5 |
| **A12** | 79 | 183 | 43 |
| **A9** | 34 | 61 | 5 |
| **A13** | 112 | 167 | 49 |
| **A14** | 27 | 35 | 1 |
| **A16** | 118 | 159 | 37 |
| **A15** | 105 | 172 | 35 |
| **B1** | 63 | 90 | 17 |
| **B2** | 67 | 66 | 19 |
| **B3** | 133 | 166 | 39 |
| **B4** | 101 | 112 | 58 |
| **B5** | 65 | 78 | 2 |
| **B6** | 85 | 171 | 25 |
| **B7** | 60 | 107 | 55 |
| **B8** | 73 | 71 | 39 |
| **B10** | 65 | 90 | 15 |
| **B11** | 130 | 172 | 15 |
| **B12** | 119 | 155 | 39 |
| **B9** | 45 | 115 | 4 |
| **B13** | 158 | 161 | 5 |
| **B14** | 70 | 98 | 79 |
| **B16** | 90 | 126 | 11 |
| **B15** | 89 | 154 | 48 |

**Color code:**

| **hepatotox** | **renal tox** |
| --- | --- |
| <50 % | <20 % |
| 50-75 % | 20-50 % |
| >75 % | >50 % |

**Cellular uptake, in vitro efficacy and in vitro toxicity of stereodefined LNA sub-libraries**

S2 - Summary of knockdown, cellular uptake, and in vitro safety profile as investigated by hepatotoxicity, renal toxicity and Caspase 3/7 activation as described in methods. In green compound sub-libraries with an improved in vitro safety profile

Improved profile


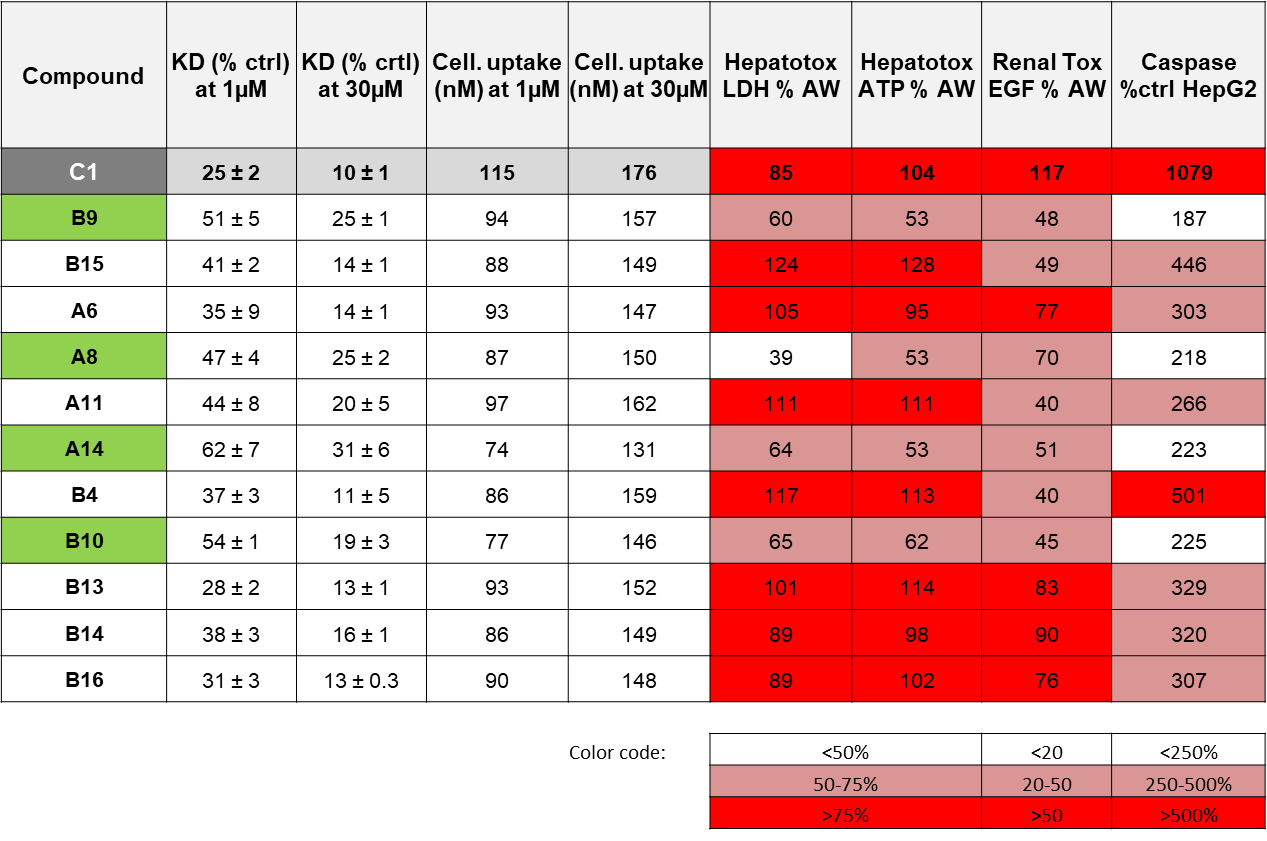


*The overall trend of reduced toxicity is reproduced in both data sets obtained. Data set on P63 includes more parameters such as cellular uptake and knock down* **Nephrotoxicity and hepatotoxicity as a function of mRNA knock down**

S3- The renal toxicity as a function of knock down in hepatocytes.

| R^2^ was calculated to 0.19 and the pearson correlation analysis gave: -0.4373 |  |
| --- | --- |

S4 - The ATP as a function of knock down in hepatocytes

|  |  |
| --- | --- |

R^2^ was calculated to 0.68 and the pearson correlation analysis gave: -0.8226

S5 - The LDH as a function of knock down in hepatocytes

|  | - |
| --- | --- |

R^2^ was calculated to 0.48 and the pearson correlation analysis gave: -0.6936

**Elaborated in vitro toxicity for figure 7**

S6 – Nephrotoxicity at 100 uM

All readouts are normalized to control oligonucleotide in screen. n = 3 for each oligonucleotide

S7 - Hepatoxicity at 30 uM (ATP)

All readouts are normalized to control oligonucleotide in screen. n = 3 for each oligonucleotide

S8 – hepatoxicity at 30 uM (LDH)

All readouts are normalized to control oligonucleotide in screen A. n = 3 for each oligonucleotide

**Statistical method used**

To investigate statistical significance we have employed the program GraphPad Prism v. 7 and GraphPad Prism v. 6.

In order to investigate if the data obtained can be determined significant we have taken advantage of the one-way analysis of variance (ANOVA) using a significance level of p < 0.05. Furthermore the Dunnett's multiple comparisons test has been used. The experiments have been done in triplicates (n = 3)

Figure 4 at 30 uM

***One-way ANOVA:***

| Table Analyzed | One-way ANOVA data | | |  |  |  |
| --- | --- | --- | --- | --- | --- | --- |
| Data sets analyzed | A : C1 30 uM | B : A8 30 uM | C : A14 30 uM | D : B9 30 uM | E : B10 30 uM | |
|  |  |  |  |  |  |  |
| ANOVA summary | |  |  |  |  |  |
| F | 18,7 |  |  |  |  |  |
| P value | 0,0001 |  |  |  |  |  |
| P value summary | *** |  |  |  |  |  |
| Significant diff. among means (P < 0.05)? | Yes |  |  |  |  |  |
| R square | 0,8821 |  |  |  |  |  |
|  |  |  |  |  |  |  |
| Brown-Forsythe test | |  |  |  |  |  |
| F (DFn, DFd) | 0,8253 (4, 10) | |  |  |  |  |
| P value | 0,5383 |  |  |  |  |  |
| P value summary | ns |  |  |  |  |  |
| Are SDs significantly different (P < 0.05)? | No |  |  |  |  |  |
|  |  |  |  |  |  |  |
| Bartlett's test | |  |  |  |  |  |
| Bartlett's statistic (corrected) | | |  |  |  |  |
| P value |  |  |  |  |  |  |
| P value summary | |  |  |  |  |  |
| Are SDs significantly different (P < 0.05)? | | | |  |  |  |
|  |  |  |  |  |  |  |
| ANOVA table | SS | DF | MS | F (DFn, DFd) | P value |  |
| Treatment (between columns) | 762,4 | 4 | 190,6 | F (4, 10) = 18,7 | P=0,0001 |  |
| Residual (within columns) | 101,9 | 10 | 10,19 |  |  |  |
| Total | 864,4 | 14 |  |  |  |  |
|  |  |  |  |  |  |  |
| Data summary | |  |  |  |  |  |
| Number of treatments (columns) | 5 |  |  |  |  |  |
| Number of values (total) | 15 |  |  |  |  |  |
|  |  |  |  |  |  |  |

***Dunnett's multiple comparisons test:***

|  |  |  |  |  |  |  |  |  |  |
| --- | --- | --- | --- | --- | --- | --- | --- | --- | --- |
| Number of families | 1 |  |  |  |  |  |  |  |  |
| Number of comparisons per family | 4 |  |  |  |  |  |  |  |  |
| Alpha | 0,05 |  |  |  |  |  |  |  |  |
|  |  |  |  |  |  |  |  |  |  |
| **Dunnett's multiple comparisons test** | **Mean Diff,** | **95,00% CI of diff,** | **Significant?** | **Summary** | **Adjusted P Value** | **A-?** |  |  |  |
|  |  |  |  |  |  |  |  |  |  |
| C1 30 uM vs. A8 30 uM | -15,67 | -23,2 to -8,132 | Yes | *** | 0,0005 | B | A8 30 uM |  |  |
| C1 30 uM vs. A14 30 uM | -21,1 | -28,63 to -13,57 | Yes | **** | <0,0001 | C | A14 30 uM | |  |
| C1 30 uM vs. B9 30 uM | -15,1 | -22,63 to -7,565 | Yes | *** | 0,0006 | D | B9 30 uM |  |  |
| C1 30 uM vs. B10 30 uM | -9,8 | -17,33 to -2,265 | Yes | * | 0,0122 | E | B10 30 uM | |  |
|  |  |  |  |  |  |  |  |  |  |
|  |  |  |  |  |  |  |  |  |  |
| Test details | Mean 1 | Mean 2 | Mean Diff, | SE of diff, | n1 | n2 | q | DF |  |
|  |  |  |  |  |  |  |  |  |  |
| C1 30 uM vs. A8 30 uM | 9,533 | 25,2 | -15,67 | 2,607 | 3 | 3 | 6,01 | 10 |  |
| C1 30 uM vs. A14 30 uM | 9,533 | 30,63 | -21,1 | 2,607 | 3 | 3 | 8,094 | 10 |  |
| C1 30 uM vs. B9 30 uM | 9,533 | 24,63 | -15,1 | 2,607 | 3 | 3 | 5,793 | 10 |  |
| C1 30 uM vs. B10 30 uM | 9,533 | 19,33 | -9,8 | 2,607 | 3 | 3 | 3,759 | 10 |  |

Figure 4 at 1 uM

***One-way ANOVA:***

| Table Analyzed | One-way ANOVA data | | |  |  |  |
| --- | --- | --- | --- | --- | --- | --- |
| Data sets analyzed | A : C1 1 uM | B : A8 1 uM | C : A14 1 uM | D : B9 1 uM | E : B10 1 uM | |
|  |  |  |  |  |  |  |
| ANOVA summary | |  |  |  |  |  |
| F | 33,11 |  |  |  |  |  |
| P value | <0,0001 |  |  |  |  |  |
| P value summary | **** |  |  |  |  |  |
| Significant diff. among means (P < 0.05)? | Yes |  |  |  |  |  |
| R square | 0,9298 |  |  |  |  |  |
|  |  |  |  |  |  |  |
| Brown-Forsythe test | |  |  |  |  |  |
| F (DFn, DFd) | 0,8955 (4, 10) | |  |  |  |  |
| P value | 0,5017 |  |  |  |  |  |
| P value summary | ns |  |  |  |  |  |
| Are SDs significantly different (P < 0.05)? | No |  |  |  |  |  |
|  |  |  |  |  |  |  |
| Bartlett's test | |  |  |  |  |  |
| Bartlett's statistic (corrected) | | |  |  |  |  |
| P value |  |  |  |  |  |  |
| P value summary | |  |  |  |  |  |
| Are SDs significantly different (P < 0.05)? | | | |  |  |  |
|  |  |  |  |  |  |  |
| ANOVA table | SS | DF | MS | F (DFn, DFd) | P value |  |
| Treatment (between columns) | 2369 | 4 | 592,2 | F (4, 10) = 33,11 | P<0,0001 |  |
| Residual (within columns) | 178,9 | 10 | 17,89 |  |  |  |
| Total | 2548 | 14 |  |  |  |  |
|  |  |  |  |  |  |  |
| Data summary | |  |  |  |  |  |
| Number of treatments (columns) | 5 |  |  |  |  |  |
| Number of values (total) | 15 |  |  |  |  |  |
|  |  |  |  |  |  |  |
|  |  |  |  |  |  |  |

***Dunnett's multiple comparisons test:***

| Number of families | 1 |  |  |  |  |  |  |  |  |
| --- | --- | --- | --- | --- | --- | --- | --- | --- | --- |
| Number of comparisons per family | 4 |  |  |  |  |  |  |  |  |
| Alpha | 0,05 |  |  |  |  |  |  |  |  |
|  |  |  |  |  |  |  |  |  |  |
| **Dunnett's multiple comparisons test** | **Mean Diff,** | **95,00% CI of diff,** | **Significant?** | **Summary** | **Adjusted P Value** | **A-?** |  |  |  |
|  |  |  |  |  |  |  |  |  |  |
| C1 1 uM vs. A8 1 uM | -22,37 | -32,35 to -12,39 | Yes | *** | 0,0003 | B | A8 1 uM |  |  |
| C1 1 uM vs. A14 1 uM | -37,6 | -47,58 to -27,62 | Yes | **** | <0,0001 | C | A14 1 uM |  |  |
| C1 1 uM vs. B9 1 uM | -26 | -35,98 to -16,02 | Yes | **** | <0,0001 | D | B9 1 uM |  |  |
| C1 1 uM vs. B10 1 uM | -29,17 | -39,15 to -19,19 | Yes | **** | <0,0001 | E | B10 1 uM |  |  |
|  |  |  |  |  |  |  |  |  |  |
|  |  |  |  |  |  |  |  |  |  |
| Test details | Mean 1 | Mean 2 | Mean Diff, | SE of diff, | n1 | n2 | q | DF |  |
|  |  |  |  |  |  |  |  |  |  |
| C1 1 uM vs. A8 1 uM | 24,53 | 46,9 | -22,37 | 3,453 | 3 | 3 | 6,477 | 10 |  |
| C1 1 uM vs. A14 1 uM | 24,53 | 62,13 | -37,6 | 3,453 | 3 | 3 | 10,89 | 10 |  |
| C1 1 uM vs. B9 1 uM | 24,53 | 50,53 | -26 | 3,453 | 3 | 3 | 7,529 | 10 |  |
| C1 1 uM vs. B10 1 uM | 24,53 | 53,7 | -29,17 | 3,453 | 3 | 3 | 8,446 | 10 |  |

**Figure 5 – mRNA knock down - details:**

***One-way ANOVA:***

| Table Analyzed | One-way ANOVA data |  |  |
| --- | --- | --- | --- |
| Data sets analyzed | A : C1 | B : B15 | C : B4 |
|  |  |  |  |
| ANOVA summary |  |  |  |
| F | 1,574 |  |  |
| P value | 0,2821 |  |  |
| P value summary | ns |  |  |
| Significant diff. among means (P < 0.05)? | No |  |  |
| R square | 0,3441 |  |  |
|  |  |  |  |
| Brown-Forsythe test |  |  |  |
| F (DFn, DFd) | 1,236 (2, 6) |  |  |
| P value | 0,3552 |  |  |
| P value summary | ns |  |  |
| Are SDs significantly different (P < 0.05)? | No |  |  |

| ANOVA table | SS | DF | MS | F (DFn, DFd) | P value |
| --- | --- | --- | --- | --- | --- |
| Treatment (between columns) | 30,59 | 2 | 15,29 | F (2, 6) = 1,574 | P=0,2821 |
| Residual (within columns) | 58,29 | 6 | 9,716 |  |  |
| Total | 88,88 | 8 |  |  |  |
|  |  |  |  |  |  |
| Data summary |  |  |  |  |  |
| Number of treatments (columns) | 3 |  |  |  |  |
| Number of values (total) | 9 |  |  |  |  |

***Dunnett's multiple comparisons test:***

| Number of families | 1 |  |  |  |  |  |  |  |
| --- | --- | --- | --- | --- | --- | --- | --- | --- |
| Number of comparisons per family | 2 |  |  |  |  |  |  |  |
| Alpha | 0,05 |  |  |  |  |  |  |  |
|  |  |  |  |  |  |  |  |  |
| **Dunnett's multiple comparisons test** | **Mean Diff,** | **95,00% CI of diff,** | **Significant?** | **Summary** | **Adjusted P Value** | **A-?** |  |  |
|  |  |  |  |  |  |  |  |  |
| C1 vs. B15 | -4,333 | -11,62 to 2,953 | No | ns | 0,2300 | B | B15 |  |
| C1 vs. B4 | -1,067 | -8,353 to 6,22 | No | ns | 0,8830 | C | B4 |  |
|  |  |  |  |  |  |  |  |  |
|  |  |  |  |  |  |  |  |  |
| Test details | Mean 1 | Mean 2 | Mean Diff, | SE of diff, | n1 | n2 | q | DF |
|  |  |  |  |  |  |  |  |  |
| C1 vs. B15 | 9,533 | 13,87 | -4,333 | 2,545 | 3 | 3 | 1,703 | 6 |
| C1 vs. B4 | 9,533 | 10,6 | -1,067 | 2,545 | 3 | 3 | 0,4191 | 6 |

**Figure 6 – details nephrotoxicity analysis:**

***One-way ANOVA:***

| Table Analyzed | One-way ANOVA data |  |  |
| --- | --- | --- | --- |
| Data sets analyzed | A : C1 | B : B15 | C : B4 |
|  |  |  |  |
| ANOVA summary |  |  |  |
| F | 36,64 |  |  |
| P value | 0,0004 |  |  |
| P value summary | *** |  |  |
| Significant diff. among means (P < 0.05)? | Yes |  |  |
| R square | 0,9243 |  |  |
|  |  |  |  |
| Brown-Forsythe test |  |  |  |
| F (DFn, DFd) | 0,1504 (2, 6) |  |  |
| P value | 0,8635 |  |  |
| P value summary | ns |  |  |
| Are SDs significantly different (P < 0.05)? | No |  |  |

| ANOVA table | SS | DF | MS | F (DFn, DFd) | P value |
| --- | --- | --- | --- | --- | --- |
| Treatment (between columns) | 10704 | 2 | 5352 | F (2, 6) = 36,64 | P=0,0004 |
| Residual (within columns) | 876,5 | 6 | 146,1 |  |  |
| Total | 11581 | 8 |  |  |  |
|  |  |  |  |  |  |
| Data summary |  |  |  |  |  |
| Number of treatments (columns) | 3 |  |  |  |  |
| Number of values (total) | 9 |  |  |  |  |

***Dunnett's multiple comparisons test:***

| \| Number of families \| 1 \|  \|  \|  \|  \|  \| \| --- \| --- \| --- \| --- \| --- \| --- \| --- \| \| Number of comparisons per family \| 2 \|  \|  \|  \|  \|  \| \| Alpha \| 0,05 \|  \|  \|  \|  \|  \| \|  \|  \|  \|  \|  \|  \|  \| \| Dunnett's multiple comparisons test \| Mean Diff, \| 95,00% CI of diff, \| Significant? \| Summary \| A-? \|  \| \|  \|  \|  \|  \|  \|  \|  \| \| C1 vs. B15 \| 67,89 \| 39,63 to 96,14 \| Yes \| *** \| B \| B15 \| \| C1 vs. B4 \| 77,48 \| 49,23 to 105,7 \| Yes \| *** \| C \| B4 \| |  |  |  |  |  |  |  |  |  |
| --- | --- | --- | --- | --- | --- | --- | --- | --- | --- | --- | --- | --- | --- | --- | --- | --- | --- | --- | --- | --- | --- | --- | --- | --- | --- | --- | --- | --- | --- | --- | --- | --- | --- | --- | --- | --- | --- | --- | --- | --- | --- | --- | --- | --- | --- | --- | --- | --- | --- | --- | --- | --- | --- | --- | --- | --- | --- | --- | --- | --- | --- | --- | --- | --- | --- |
|  |  |  |  |  |  |  |  |  |  |
| \| Test details \| Mean 1 \| Mean 2 \| Mean Diff, \| SE of diff, \| n1 \| n2 \| q \| DF \| \| --- \| --- \| --- \| --- \| --- \| --- \| --- \| --- \| --- \| \|  \|  \|  \|  \|  \|  \|  \|  \|  \| \| C1 vs. B15 \| 117,3 \| 49,44 \| 67,89 \| 9,869 \| 3 \| 3 \| 6,879 \| 6 \| \| C1 vs. B4 \| 117,3 \| 39,85 \| 77,48 \| 9,869 \| 3 \| 3 \| 7,851 \| 6 \| |  |  |  |  |  |  |  |  |  |
|  |  |  |  |  |  |  |  |  |  |
|  |  |  |  |  |  |  |  |  |  |
|  |  |  |  |  |  |  |  |  |  |
| Cell uptake analysis  At 30 uM:  ***One-way ANOVA:*** |  |  |  |  |  |  |  |  |  |
| \| Table Analyzed \| Data 1 \|  \|  \|  \|  \|  \| \| --- \| --- \| --- \| --- \| --- \| --- \| --- \| \| Data sets analyzed \| A : B15 \| B : A8 \| C : B9 \| D : A6 \| E : A11 \|  \| \|  \|  \|  \|  \|  \|  \|  \| \| ANOVA summary \| \|  \|  \|  \|  \|  \| \| F \| 5,106 \|  \|  \|  \|  \|  \| \| P value \| 0,0004 \|  \|  \|  \|  \|  \| \| P value summary \| *** \|  \|  \|  \|  \|  \| \| Significant diff. among means (P < 0.05)? \| Yes \|  \|  \|  \|  \|  \| \| R square \| 0,7006 \|  \|  \|  \|  \|  \| \|  \|  \|  \|  \|  \|  \|  \| \| Brown-Forsythe test \| \|  \|  \|  \|  \|  \| \| F (DFn, DFd) \| 0,3167 (11, 24) \| \|  \|  \|  \|  \| \| P value \| 0,9746 \|  \|  \|  \|  \|  \| \| P value summary \| ns \|  \|  \|  \|  \|  \| \| Are SDs significantly different (P < 0.05)? \| No \|  \|  \|  \|  \|  \| \|  \|  \|  \|  \|  \|  \|  \| \| Bartlett's test \| \|  \|  \|  \|  \|  \| \| Bartlett's statistic (corrected) \| \| \|  \|  \|  \|  \| \| P value \|  \|  \|  \|  \|  \|  \| \| P value summary \| \|  \|  \|  \|  \|  \| \| Are SDs significantly different (P < 0.05)? \| \| \| \|  \|  \|  \| \|  \|  \|  \|  \|  \|  \|  \| \| ANOVA table \| SS \| DF \| MS \| F (DFn, DFd) \| P value \|  \| \| Treatment (between columns) \| 3421 \| 11 \| 311 \| F (11, 24) = 5,106 \| P=0,0004 \|  \| \| Residual (within columns) \| 1462 \| 24 \| 60,92 \|  \|  \|  \| \| Total \| 4883 \| 35 \|  \|  \|  \|  \| \|  \|  \|  \|  \|  \|  \|  \| \| Data summary \| \|  \|  \|  \|  \|  \| \| Number of treatments (columns) \| 12 \|  \|  \|  \|  \|  \| \| Number of values (total) \| 36 \|  \|  \|  \|  \|  \|   ***One-way ANOVA:*** |  |  |  |  |  |  |  |  |  |
|  |  |  |  |  |  |  |  |  |  |
|  |  |  |  |  |  |  |  |  |  |
|  |  |  |  |  |  |  |  |  |  |
|  |  |  |  |  |  |  |  |  |  |
|  |  |  |  |  |  |  |  |  |  |
|  |  |  |  |  |  |  |  |  |  |

***Dunnett's multiple comparisons test:***

| Number of families | 1 |  |  |  |  |  |  |  |  |
| --- | --- | --- | --- | --- | --- | --- | --- | --- | --- |
| Number of comparisons per family | 11 |  |  |  |  |  |  |  |  |
| Alpha | 0,05 |  |  |  |  |  |  |  |  |
|  |  |  |  |  |  |  |  |  |  |
| Dunnett's multiple comparisons test | Mean Diff, | 95,00% CI of diff, | Significant? | Summary | Adjusted P Value | L-? |  |  |  |
|  |  |  |  |  |  |  |  |  |  |
| C1 vs. B15 | 19 | 0,08747 to 37,91 | Yes | * | 0,0486 | A | B15 |  |  |
| C1 vs. A8 | 29,33 | 10,42 to 48,25 | Yes | ** | 0,001 | B | A8 |  |  |
| C1 vs. B9 | 23,67 | 4,754 to 42,58 | Yes | ** | 0,0091 | C | B9 |  |  |
| C1 vs. A6 | 26 | 7,087 to 44,91 | Yes | ** | 0,0038 | D | A6 |  |  |
| C1 vs. A11 | 14 | -4,913 to 32,91 | No | ns | 0,2288 | E | A11 |  |  |
| C1 vs. B4 | 17,33 | -1,579 to 36,25 | No | ns | 0,0843 | F | B4 |  |  |
| C1 vs. A14 1 uM | 41,67 | 22,75 to 60,58 | Yes | **** | <0,0001 | G | A14 1 uM |  |  |
| C1 vs. B10 1 uM | 30 | 11,09 to 48,91 | Yes | *** | 0,0008 | H | B10 1 uM |  |  |
| C1 vs. B13 | 24 | 5,087 to 42,91 | Yes | ** | 0,0081 | I | B13 |  |  |
| C1 vs. B16 | 27,67 | 8,754 to 46,58 | Yes | ** | 0,002 | J | B16 |  |  |
| C1 vs. B14 | 26,67 | 7,754 to 45,58 | Yes | ** | 0,0029 | K | B14 |  |  |
|  |  |  |  |  |  |  |  |  |  |
|  |  |  |  |  |  |  |  |  |  |
| Test details | Mean 1 | Mean 2 | Mean Diff, | SE of diff, | n1 | n2 | q | DF |  |
|  |  |  |  |  |  |  |  |  |  |
| C1 vs. B15 | 176 | 157 | 19 | 6,373 | 3 | 3 | 2,981 | 24 |  |
| C1 vs. A8 | 176 | 146,7 | 29,33 | 6,373 | 3 | 3 | 4,603 | 24 |  |
| C1 vs. B9 | 176 | 152,3 | 23,67 | 6,373 | 3 | 3 | 3,714 | 24 |  |
| C1 vs. A6 | 176 | 150 | 26 | 6,373 | 3 | 3 | 4,08 | 24 |  |
| C1 vs. A11 | 176 | 162 | 14 | 6,373 | 3 | 3 | 2,197 | 24 |  |
| C1 vs. B4 | 176 | 158,7 | 17,33 | 6,373 | 3 | 3 | 2,72 | 24 |  |
| C1 vs. A14 1 uM | 176 | 134,3 | 41,67 | 6,373 | 3 | 3 | 6,538 | 24 |  |
| C1 vs. B10 1 uM | 176 | 146 | 30 | 6,373 | 3 | 3 | 4,708 | 24 |  |
| C1 vs. B13 | 176 | 152 | 24 | 6,373 | 3 | 3 | 3,766 | 24 |  |
| C1 vs. B16 | 176 | 148,3 | 27,67 | 6,373 | 3 | 3 | 4,341 | 24 |  |
| C1 vs. B14 | 176 | 149,3 | 26,67 | 6,373 | 3 | 3 | 4,185 | 24 |  |

At 1 uM

***One-way ANOVA:***

| Table Analyzed | Data 1 |  |  |  |  |  |  |
| --- | --- | --- | --- | --- | --- | --- | --- |
| Data sets analyzed | A : B15 | B : A8 | C : B9 | D : A6 | E : A11 |  |  |
|  |  |  |  |  |  |  |  |
| ANOVA summary | |  |  |  |  |  |  |
| F | 8,331 |  |  |  |  |  |  |
| P value | <0,0001 |  |  |  |  |  |  |
| P value summary | **** |  |  |  |  |  |  |
| Significant diff. among means (P < 0.05)? | Yes |  |  |  |  |  |  |
| R square | 0,7925 |  |  |  |  |  |  |
|  |  |  |  |  |  |  |  |
| Brown-Forsythe test | |  |  |  |  |  |  |
| F (DFn, DFd) | 0,332 (11, 24) | |  |  |  |  |  |
| P value | 0,9698 |  |  |  |  |  |  |
| P value summary | ns |  |  |  |  |  |  |
| Are SDs significantly different (P < 0.05)? | No |  |  |  |  |  |  |
|  |  |  |  |  |  |  |  |
| Bartlett's test | |  |  |  |  |  |  |
| Bartlett's statistic (corrected) | | |  |  |  |  |  |
| P value |  |  |  |  |  |  |  |
| P value summary | |  |  |  |  |  |  |
| Are SDs significantly different (P < 0.05)? | | | |  |  |  |  |
|  |  |  |  |  |  |  |  |
| ANOVA table | SS | DF | MS | F (DFn, DFd) | P value |  |  |
| Treatment (between columns) | 3541 | 11 | 321,9 | F (11, 24) = 8,331 | P<0,0001 |  |  |
| Residual (within columns) | 927,3 | 24 | 38,64 |  |  |  |  |
| Total | 4468 | 35 |  |  |  |  |  |
|  |  |  |  |  |  |  |  |
| Data summary | |  |  |  |  |  |  |
| Number of treatments (columns) | 12 |  |  |  |  |  |  |
| Number of values (total) | 36 |  |  |  |  |  |  |
|  |  |  |  |  |  |  |  |

***Dunnett's multiple comparisons test:***

| Number of families | 1 |  |  |  |  |  |  |  |  |
| --- | --- | --- | --- | --- | --- | --- | --- | --- | --- |
| Number of comparisons per family | 11 |  |  |  |  |  |  |  |  |
| Alpha | 0,05 |  |  |  |  |  |  |  |  |
|  |  |  |  |  |  |  |  |  |  |
| Dunnett's multiple comparisons test | Mean Diff, | 95,00% CI of diff, | Significant? | Summary | Adjusted P Value | L-? |  |  |  |
|  |  |  |  |  |  |  |  |  |  |
| C1 vs. B15 | 20,67 | 5,604 to 35,73 | Yes | ** | 0,0039 | A | B15 |  |  |
| C1 vs. A8 | 21 | 5,938 to 36,06 | Yes | ** | 0,0033 | B | A8 |  |  |
| C1 vs. B9 | 26,67 | 11,6 to 41,73 | Yes | *** | 0,0002 | C | B9 |  |  |
| C1 vs. A6 | 28,67 | 13,6 to 43,73 | Yes | **** | <0,0001 | D | A6 |  |  |
| C1 vs. A11 | 18 | 2,938 to 33,06 | Yes | * | 0,0136 | E | A11 |  |  |
| C1 vs. B4 | 28,33 | 13,27 to 43,4 | Yes | *** | 0,0001 | F | B4 |  |  |
| C1 vs. A14 | 41 | 25,94 to 56,06 | Yes | **** | <0,0001 | G | A14 |  |  |
| C1 vs. B10 | 37,67 | 22,6 to 52,73 | Yes | **** | <0,0001 | H | B10 |  |  |
| C1 vs. B13 | 22 | 6,938 to 37,06 | Yes | ** | 0,002 | I | B13 |  |  |
| C1 vs. B16 | 24,33 | 9,271 to 39,4 | Yes | *** | 0,0007 | J | B16 |  |  |
| C1 vs. B14 | 29,33 | 14,27 to 44,4 | Yes | **** | <0,0001 | K | B14 |  |  |
|  |  |  |  |  |  |  |  |  |  |
|  |  |  |  |  |  |  |  |  |  |
| Test details | Mean 1 | Mean 2 | Mean Diff, | SE of diff, | n1 | n2 | q | DF |  |
|  |  |  |  |  |  |  |  |  |  |
| C1 vs. B15 | 114,7 | 94 | 20,67 | 5,075 | 3 | 3 | 4,072 | 24 |  |
| C1 vs. A8 | 114,7 | 93,67 | 21 | 5,075 | 3 | 3 | 4,138 | 24 |  |
| C1 vs. B9 | 114,7 | 88 | 26,67 | 5,075 | 3 | 3 | 5,254 | 24 |  |
| C1 vs. A6 | 114,7 | 86 | 28,67 | 5,075 | 3 | 3 | 5,648 | 24 |  |
| C1 vs. A11 | 114,7 | 96,67 | 18 | 5,075 | 3 | 3 | 3,547 | 24 |  |
| C1 vs. B4 | 114,7 | 86,33 | 28,33 | 5,075 | 3 | 3 | 5,583 | 24 |  |
| C1 vs. A14 | 114,7 | 73,67 | 41 | 5,075 | 3 | 3 | 8,078 | 24 |  |
| C1 vs. B10 | 114,7 | 77 | 37,67 | 5,075 | 3 | 3 | 7,421 | 24 |  |
| C1 vs. B13 | 114,7 | 92,67 | 22 | 5,075 | 3 | 3 | 4,335 | 24 |  |
| C1 vs. B16 | 114,7 | 90,33 | 24,33 | 5,075 | 3 | 3 | 4,794 | 24 |  |
| C1 vs. B14 | 114,7 | 85,33 | 29,33 | 5,075 | 3 | 3 | 5,78 | 24 |  |
